# Role of *Klebsiella pneumoniae* Type VI secretion system (T6SS) in long-term gastrointestinal colonization

**DOI:** 10.1038/s41598-022-21396-w

**Published:** 2022-10-10

**Authors:** Thomas Merciecca, Stéphanie Bornes, Laurence Nakusi, Sébastien Theil, Olaya Rendueles, Christiane Forestier, Sylvie Miquel

**Affiliations:** 1 Université Clermont Auvergne, CNRS, LMGE, 63000 Clermont–Ferrand, France; 2 Université Clermont Auvergne, INRAE, VetAgro Sup, UMRF, 63000 Clermont–Ferrand, France; 3 Institut Pasteur, Université de Paris, CNRS, UMR3525, Microbial Evolutionary Genomics, 75015 Paris, France

**Keywords:** Microbiome, Bacteriology

## Abstract

Type VI secretion systems (T6SS), recently described in hypervirulent *K. pneumoniae* (hvKp) strains*,* are involved in bacterial warfare but their role in classical clinical strains (cKp) has been little investigated*. *In silico analysis indicated the presence of T6SS clusters (from zero to four), irrespective of the strains origin or virulence, with a high prevalence in the *K. pneumoniae* species (98%). In the strain CH1157, two T6SS-apparented pathogenicity islands were detected, T6SS-1 and -2, harboring a phospholipase-encoding gene (*tle1*) and a potential new effector-encoding gene named *tke* (Type VI *Klebsiella* effector). Tle1 expression in *Escherichia coli* periplasm affected cell membrane permeability. T6SS-1 isogenic mutants colonized the highest gastrointestinal tract of mice less efficiently than their parental strain, at long term. Comparative analysis of faecal 16S sequences indicated that T6SS-1 impaired the microbiota richness and its resilience capacity. Oscillospiraceae family members could be specific competitors for the long-term gut establishment of *K. pneumoniae*.

## Introduction

*Klebsiella pneumoniae* is an opportunistic Gram-negative pathogen that is considered by the World Health Organization (WHO) and the Center for Disease Control and Prevention (CDC) to be a major health threat. Classical clinical isolates are responsible for hospital-acquired infections such as urinary tract infections, pneumonia, meningitis and endophthalmitis. *K. pneumoniae* is responsible for severe disseminated infections after broad-spectrum antibiotic treatment^1^, partly due to its intrinsic resistance to β-lactam antibiotics combined with recently acquired resistance to carbapenems^2,3^. Hypervirulent *K. pneumoniae* (hvKp) are also responsible for severe community-acquired infections and are particularly associated with pyogenic liver abscesses^4–6^. Their enhanced virulence is due to the overproduction of factors such as siderophores and capsular polysaccharides. The hypervirulence phenotype is linked to the presence of a plasmid (pLVPK) that harbors several genes or operons (*rmpA*, *magA* and *iro* and *iuc* operons) involved in the synthesis of virulence factors^7–10^. *K. pneumoniae* is able to survive in different environments such as soil, plants, dust, and water^11,12^, and human infections are usually preceded by gastro-intestinal colonization^1^. The mechanisms allowing *K. pneumoniae* to establish within the intestinal microbiota are poorly understood but they are likely to require a competitive advantage over resident intestinal microbiota^13^.

In Gram-negative pathogens, secretion systems are involved in bacterial fitness^14–16^. The Type VI Secretion System (T6SS) was initially described in *Vibrio cholerae* as an extracellular protein translocation system^17^ and has since been largely documented in Bacteroidetes and Proteobacteria, especially in Enterobacteriaceae^18^. It consists of a molecular syringe functionally and structurally related to the contractile tail of bacteriophages^19^. T6SS apparatus is composed of three major subunits: a periplasmic anchored baseplate complex, an inner syringe made up of Hcp protein hexamers fixed to a VgrG/PAAR protein tip complex acting like a puncture device. Once fully assembled, the TssBC contractile tube sheath engulfs the inner syringe leading to the T6SS effector extrusion. After contraction, an ATPase, ClpV, is engaged to recycle the different subunits allowing further attacks^20^. Bacteria harboring T6SS are thus able to inject toxic effectors in adjacent cells via direct contact leading to the death of preys^21^. Different kinds of effectors have been described according to their mode of action including DNAse, phospholipase, glycoside, and hydrolase^22^. Each toxic effector has an associated cognate immunity protein that prevents T6SS-carrying bacteria from self-intoxication and acts as a paired toxin-antitoxin system^22,23^. The presence of T6SS confers an advantage in highly competitive environments such as host gut microbiota^24,25^. For instance, the intestinal pathogen *Salmonella* Typhimurium T6SS provides antibacterial activity against specific gut microbiota individuals such as *K. oxytoca* and *K. variicola*, suggesting that it kills microbiota members to invade the mice gut^26^. *Shigella sonei* is also able to use its T6SS to eliminate gut competitors in vivo (*E. coli* and *Shigella flexneri*) in mice^27^.

Sarris et al. provided first evidence of T6SS in *K. pneumoniae* species in 2011^28^. The first *K. pneumoniae* T6SS effector, a phospholipase (Pld1), was then identified and its role in pulmonary infection was demonstrated in a murine model using a *pld1* deletion mutant strain^29^. More recently, analysis of 107 fully sequenced genomes of *K. pneumoniae* identified 254 T6SS related clusters^30^, and their presence was positively correlated with the hypervirulent phenotype^31,32^. T6SS are conserved worldwide across the genome of *K. pneumoniae* sequence type 258 (ST258) causing health-care-associated pneumonia^33^. In vitro intra- and inter-species antibacterial activities have been associated with T6SS expression, and a phospholipase (Tle1^KP^) was identified as the effector of the multidrug resistant strain HS11286 and a toxin-antitoxin system including a phospholipase effector (Tle1^KP^) and its cognate immunity protein (Tli1^KP^) was identified in the multidrug resistant strain HS11286, conferring Tle1^KP^ resistance^30^. Other phospholipase T6SS effectors have already been identified in *K. pneumoniae* species (Tle3)^34^, but due to the diversity of T6SS effectors^35,36^ others remain to be identified.

Most data so far have been obtained with hvKp isolates, and the prevalence and function of T6SS in classical *K. pneumoniae* (cKp) strains have not been fully investigated despite the strains being capable of colonizing the intestinal tract. In this study, an in silico approach was used to investigate the prevalence of T6SS and its effectors among documented *Klebsiella* database. The cKp CH1157 strain was used to investigate the toxic effect of Tle1 in vitro using site-directed *Escherichia coli* toxicity assays. In addition, intestinal colonization assays were performed in a murine model to assess the role of T6SS-1 in colonization kinetics of the intestinal tract and its impact on intestinal microbiota composition via 16S rRNA high-throughput sequencing.

## Results

### Prevalence of T6SS clusters among *Klebsiella* genus and genome organization of T6SS islands in CH1157 strain

**Figure 1:**
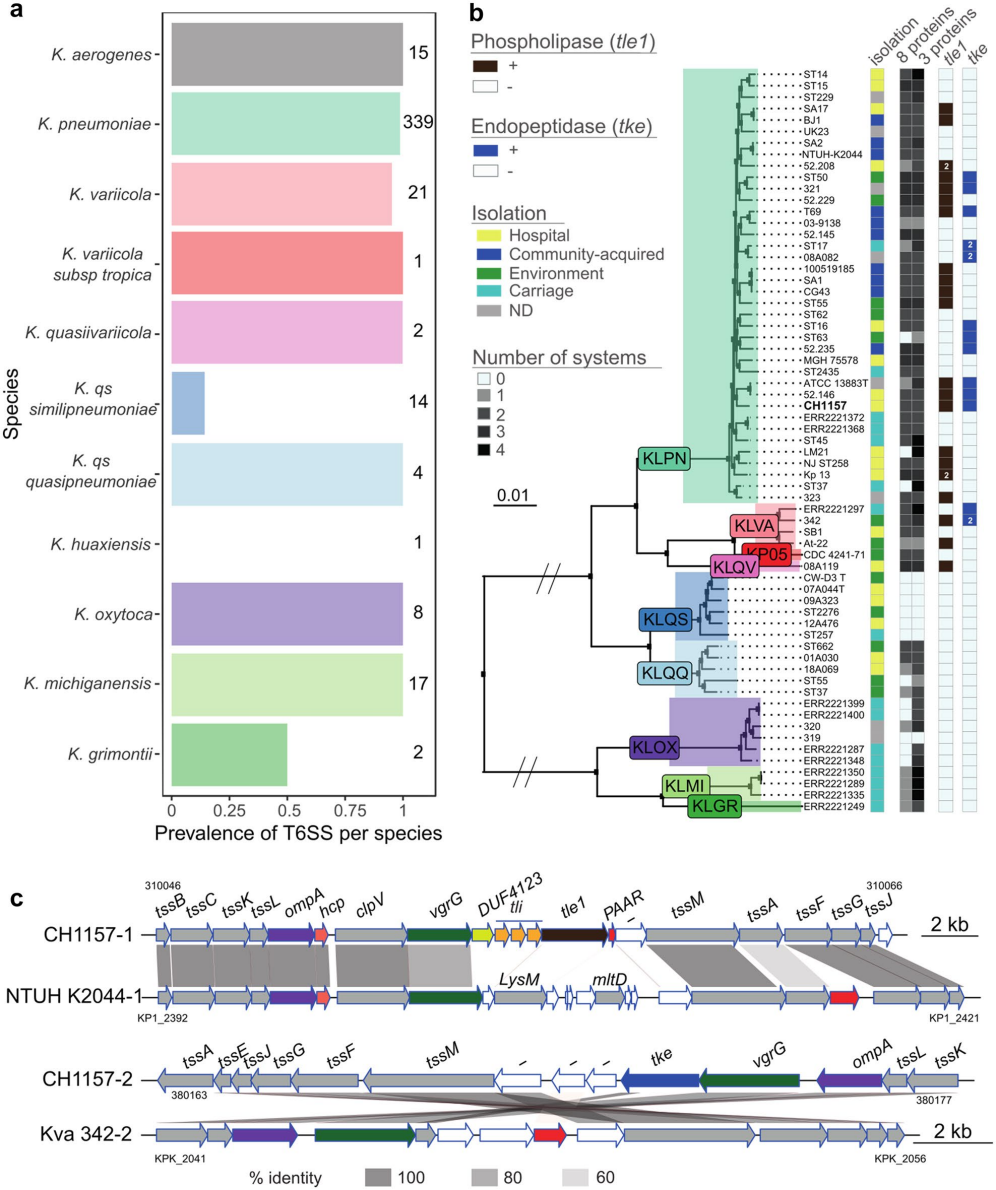
Distribution and characterization of Type VI secretion systems across the *Klebsiella* genus. **(a)** Prevalence of T6SS is calculated as the ratio of genomes of each species with at least one complete T6SS. Numbers represent the total number of genomes analyzed per species. (**b**) Unrooted tree built using 2969 families of the persistent genome of the representative strains of *Klebsiella* genus. The first column determines the environment from which the strains were isolated (ND not determined). The second and third columns report the number of T6SS per strain using a minimum number of 3 or 8 proteins, respectively, to consider the presence of a T6SS cluster. The last two columns indicate the presence or absence of phospholipase-encoding and endopeptidase-encoding genes (with at least 80% and 50% identity to that found in T6SSs clusters, respectively). The tree was built using the packages *ggtree* and *treeio* for R. KLPN : *K. pneumoniae* ; KLVA : *K. variicola* ; KP05 : *K. pneumoniae 05* ; KLQV : *K. quasiivariicola* ; KLQS : *K. qs similipneumoniae* ; KLQQ : *K. qs quasipneumoniae* ; KLOX : *K. oxytoca* ; KLMI : *K. michiganensis* ; KLGR : *K. grimontii* **(c)** Diagram of the two clusters of CH1157 strain, one of strain NTUH-K2044 and another of *K. variicola* 342. White arrows indicate unknown function, and gray arrows indicate genes that are core to the T6SS. The diagram was built with the *genoplotR* package^64^*.* The identity percentages were calculated using blastp (v BLAST 2.7.1+, default parameters).

The prevalence of T6SS in *Klebsiella* genomes was assessed using MacSyFinder^37^ and custom-made T6SS models^38^ within 423 complete genomes of the *Klebsiella* genus of which 339 belonged to *K. pneumoniae* and 22 to *K. variicola* (Supplementary Table S1a). Both *K. pneumoniae* and *K. variicola* had a high prevalence of T6SS clusters, with 98% (332 out of 339) and 95% (20 out of 21) of the strains harboring at least one T6SS, respectively (Fig. 1a). T6SS was present in a remarkably low proportion of *K. quasi subsp similipneumoniae* representatives (2 out of 14). To further investigate this distribution, we downloaded 45 other complete genomes of *K. quasi subsp similpneumoniae* (Supplementary Table S1b). Their inclusion did not significantly alter the results, with a global prevalence of 0.2 (in 12 out of the 59 analysed). On average, 1.99 T6SS clusters per genome were found, ranging from 0 to 4 across genomes considering the presence of at least 8 different clustered proteins among TssA, TssB, TssC, TssD (Hcp), TssE, TssF, TssG, TssH (ClpV), TssI (VgrG), TssJ, TssK, TssL, TssM and EvpJ (PAAR) (Fig. 1b, Supplementary Fig. S1). Decreasing the number of proteins required for T6SS systems to be considered complete increased the number of T6SS clusters in genomes that already had a complete T6SS (Supplementary Fig. S2b and c). Accordingly, genomes without a full system (by considering 8 core proteins) did not harbor incomplete T6SS operons (Supplementary Fig. S2b and c).

No significant association was found between the presence of *rmpA* (a capsule regulator involved in hypermucoid phenotype and a proxy of hypervirulence) and the number of T6SS clusters in a genome (Kruskal–Wallis Test, *P* = 0.7) (Supplementary Table S1a). Similarly, no association was observed between the number of T6SS clusters and the virulence score of *Klebsiella* (Spearman’s test, *P* = 0.7) (Supplementary Table S1a). Using a smaller database (KL65) representative of the diversity of *Klebsiella* and curated for strain origin (see methods) evidenced no significant association between the origin of the strain and the prevalence of T6SS (Fisher’s Exact Test, *P* = 0.13) (Fig. 1b**)**.

In the laboratory model strain CH1157, two T6SS-apparented pathogenicity islands were detected, hereafter referred to as T6SS-1 and -2 (27 and 21 kb, respectively). T6SS-1 is closely related to T6SS-1 of reference hypervirulent NTUH-K2044 strain owing their genomic organization and the synteny between the two T6SS core gene loci (Fig. 1c). In contrast, the regions between *vgrG* and *tssM* (corresponding to the toxin-antitoxin system) are highly specific to each strain and likely possess specific T6SS dependent effector encoding genes. T6SS-1 of strain CH1157 encompasses a potentially phospholipase encoding gene (*tle1*) comprising a DUF2235 domain and its cognate three antitoxin genes named *tli1* whereas NTUH-K2044 possesses a LysM gene with a peptidoglycan binding domain and an MltD gene (murein lytic transglycosylase). In the KL65 database, the presence of Tle1 phospholipase toxic effector seems to be specific to the *K. pneumoniae* species complex, since it was detected only in 18 *K. pneumoniae* strains, 2 *K. variicola* strains (342 and At-22) and 1 *K. quasivariicola* (Fig. 1b).

The CH1157 T6SS-2 cluster differs slightly from T6SS-1, having fewer core genes (*tssBC, hcp*, *PAAR*, *clpV* missing) and a different cluster organization. However, synteny was observed between T6SS-2 locus and the second T6SS cluster of *K. variicola* 342 **(**Fig. 1c). The effector in CH1157 T6SS-2 was different from that of T6SS-1 and potentially encodes an endopeptidase M23-like protein. This hypothetical protein comprises an N-Terminal LysM peptidoglycan binding domain (PF 01476.23), a SLT transglycosylase domain (PF 01464.23), and a C-Terminal M23 peptidase family (PF 01551.25). The prevalence of such a M23-like protein was investigated in silico in 13514 complete genomes from 4232 different bacterial species **(**Supplementary Fig. S3). The presence of this protein seems to be exclusive to *Klebsiella* genus and *K. pneumoniae* species and was thus named “Tke” (Type VI *Klebsiella* Effector) following the nomenclature of T6SS bacterial effectors (Fig. 1b). No cognate immunity protein was identified.

### Membrane permeability associated with periplasmic expression of Tle1 phospholipase

**Figure 2:**
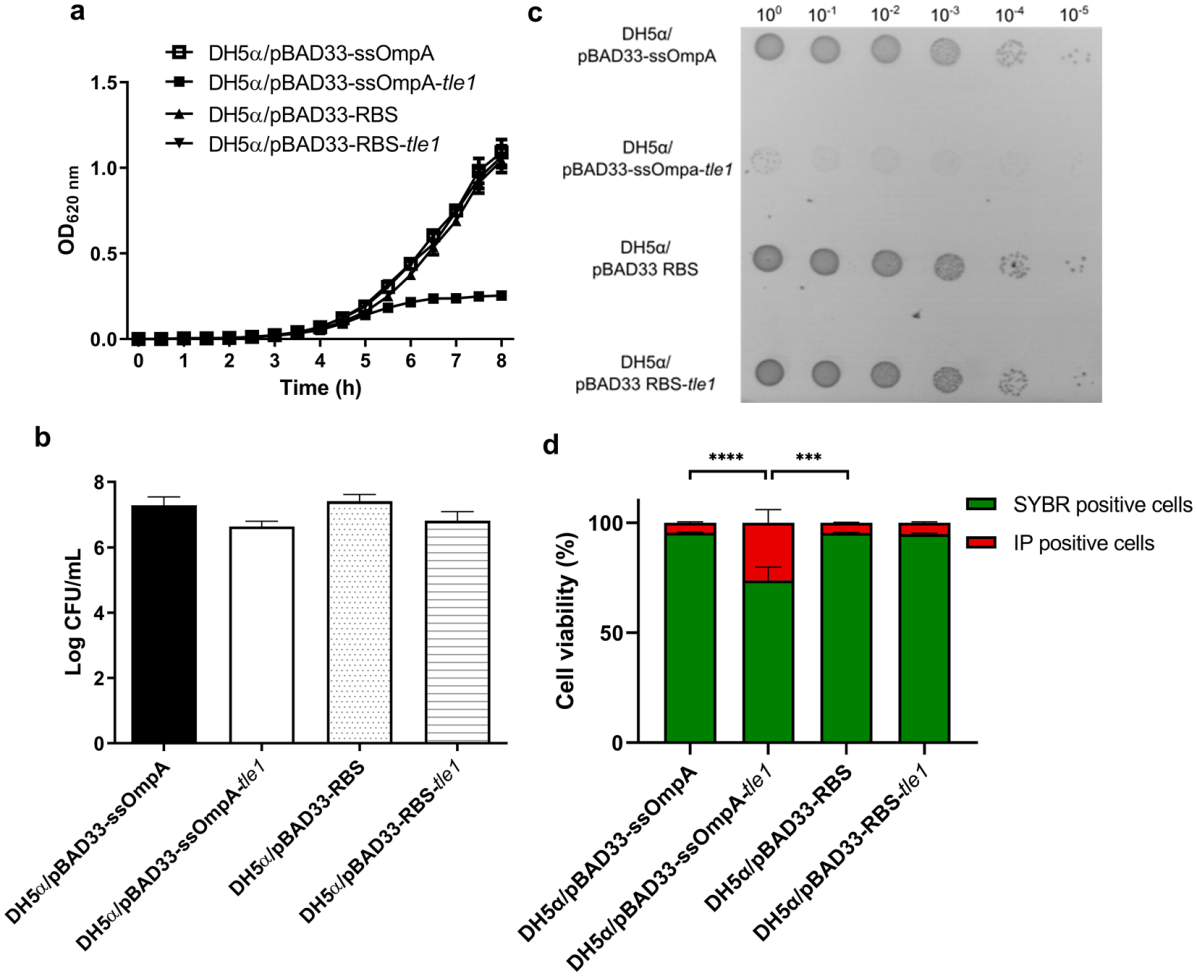
Characterization of Tle1 phospholipase of T6SS-I of CH1476. **(a)** Growth curves of *E. coli* DH5α harboring pBAD33 derived constructions. **(b)** CFU counts at 8 h of culture of *E. coli* DH5α strains carrying pBAD33-derived constructions. **(c**) Toxicity assays performed on 0.2% arabinose-containing LB agar plates. Pictures are representative of three independent experiments. **(d**) SYBR/IP stained-cells ratio of *E. coli* DH5α strains carrying pBAD33-derived constructions. Data are expressed as means ± SEM (N = 9). *P*-values were derived from comparisons of each strain carrying pBAD33 with *tle1* gene copy with cognate native pBAD33 plasmid or between *tle1* containing pBAD33-ssOmpA and pBAD33-RBS strains via nonparametric Kruskal–Wallis and Dunn’s multiple comparison test: *P < 0.05, **P < 0.01, ***P < 0.005, ****P < 0.001.

The Tle1 effector antibacterial effect has been previously reported^30^. To assess if this phenotype was associated with the effector of strain CH1157, heterologous expression of Tle1 in *E. coli* DH5α was performed. Addressing of Tle1 specifically to the periplasm significantly affected bacterial growth, as evidenced by spectrometric measurement of the growth of *E. coli* DH5α/pBAD33-ssOmpA-*tle1* compared to that of the control strain (*E. coli* DH5α/pBAD33-ssOmpA) (Fig. 2a). In contrast, cytoplasmic addressing of Tle1 (*E. coli* DH5α recombinant strain harboring pBAD33-RBS-*tle1*) did not affect bacterial growth compared to that of the control strain (*E. coli* DH5α/pBAD33-RBS). Determination of the number of viable cells at the final point (8 h) did not show any significant differences between the four strains **(**Fig. 2b) but an unusual microcolony phenotype was associated with periplasmic addressing of Tle1 (Fig. 2c). Flow cytometry analysis after SYBR Green and propidium iodide (IP) staining showed that when Tle1 was located in the periplasm it significantly enhanced cell membrane permeability compared to Tle1 in the cytoplasm and DH5α control strains (DH5α/pBAD33-ssOmpA and DH5α/pBAD33-RBS) (Fig. 2d).

### *K. pneumoniae* T6SS-1 is involved in mice intestinal colonization

**Figure 3:**
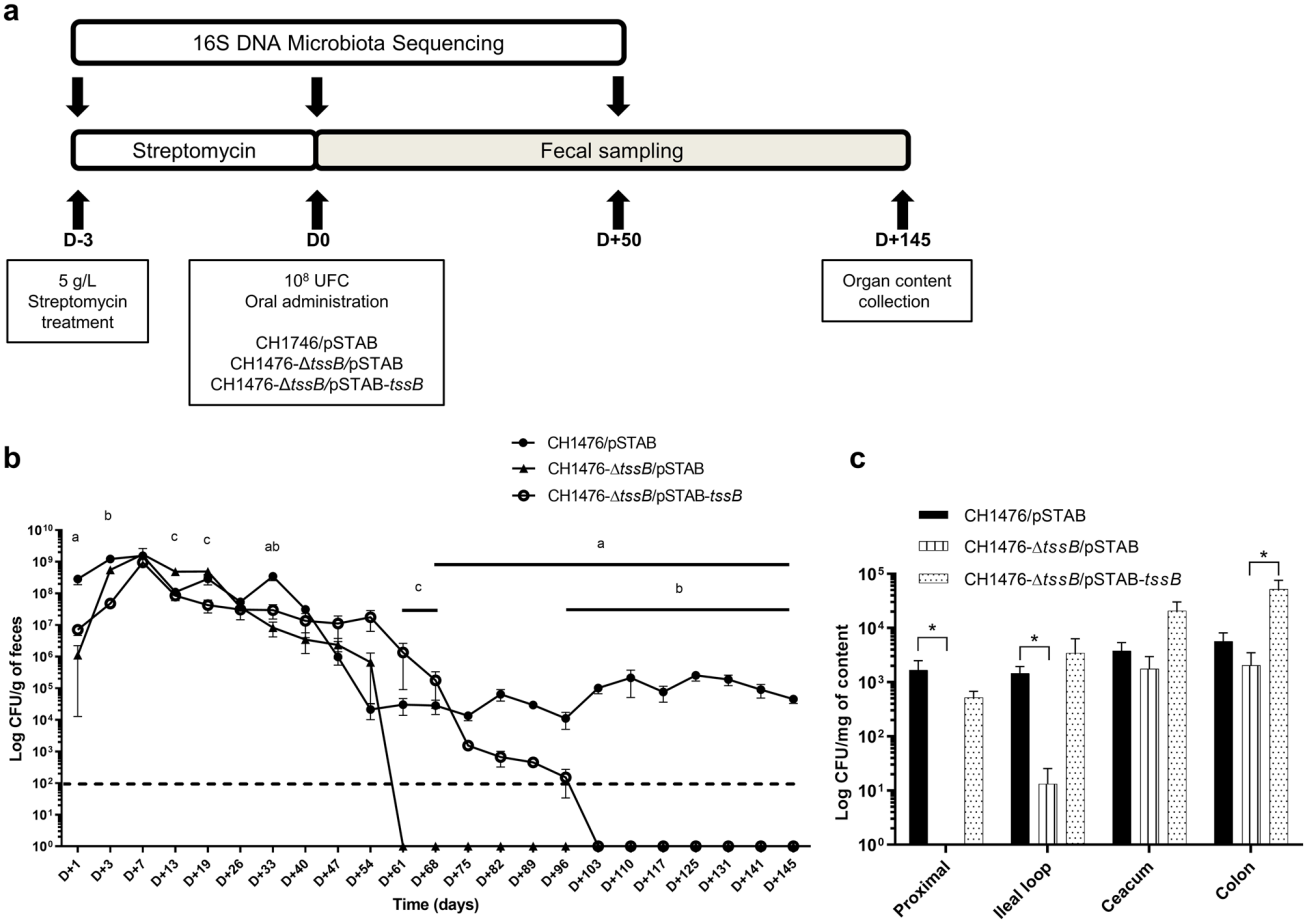
Role of T6SS-1 in gastrointestinal colonization of mice by *K. pneumoniae* CH1476. **(a)** Experimental procedure (n = 6/group). **(b)** After 72 h of streptomycin treatment (5 g/L in drinking water), mice were intragastrically inoculated with 1 × 10^8^ CFU of *K. pneumoniae* CH1476 strain (CH1476/pSTAB, full circle), CH1476-Δ*tssB* isogenic mutant (CH1476-Δ*tssB*/pSTAB, full triangle) or the trans-complemented control strain (CH1476-Δ*tssB*/pSTAB-*tssB*, empty circle). Results are expressed as means *K. pneumoniae* CFU/g of feces ± SEM over 145 days; the dashed line indicates the limit of detection (10^2^ CFU). Statistical analysis: nonparametric Kruskall-Wallis with Dunn’s multiple comparison test: a, b, c: p < 0.05, respectively CH1476/pSTAB vs CH1476-Δ*tssB*/pSTAB, CH1476/pSTAB vs CH1476-Δ*tssB*/pSTAB-*tssB* and CH1476-Δ*tssB*/pSTAB vs CH1476-Δ*tssB*/pSTAB-*tssB*. **(c)** Representation along the gastrointestinal tract of *K. pneumoniae* CH1476/pSTAB (black bar), CH1476-Δ*tssB*/pSTAB isogenic mutant (full line bar) or the CH1476-Δ*tssB*/pSTAB-*tssB* trans-complemented control strain (dotted line bar) in CFU/g of content in mice. Data are expressed as means of each 6 values ± SEM. Statistical analysis: nonparametric Kruskall-Wallis with Dunn’s multiple comparison test: *P < 0.05.

In absence of streptomycin treatment, the colonization of the gut by *K. pneumoniae* CH1476 (a fully virulent CH1157-derived strain^39^ (see Material and Methods)) could not be followed in feces due to its very low implantation until 10 days post- infection (5.21.10^2^ ± 2.17. 10^2^ CFU/g of feces; detection threshold: 10^2^ CFU/g of feces). In the streptomycin-treated murine model, whatever the strain tested (CH1476 wild-type harboring the empty vector, the isogenic mutant CH1476-Δ*tssB*/pSTAB or the trans-complemented strain CH1476-Δ*tssB*/pSTAB-*tssB*) (Fig. 3a), a peak of colonization level was reached after 7 days of infection (10^9^ UFC/g of feces), followed by stable colonization kinetics until 54 days post-infection (Fig. 3b).

CH1476-Δ*tssB*/pSTAB mutant strain then became undetectable in the stool 61 days post-infection while the CH1476-Δ*tssB*/pSTAB-*tssB* trans-complemented and CH1476/pSTAB WT strains remained detectable until 96 and 145 days post-infection, respectively. Analysis of the intestinal content of the three sets of mice on day 145 post-infection showed that CH1476-Δ*tssB*/pSTAB was mainly present in the lower part of the gastrointestinal tract, whereas the CH1476/pSTAB WT and the trans-complemented strains were detected at similar levels in the proximal intestine and the colon (between 10^3^ and 10^4^ CFU/g of content) (Fig. 3c). Similar results were obtained with the CH1476-Δ*clpV* mutant, which is deficient in T6SS recycling. CH1476-Δ*clpV* strain colonization levels were significantly lower than those of the CH1476 parental strain 80 days after infection (3.1.10^4^ CFU/g of feces vs 8.5.10^6^ CFU/g of feces respectively). In addition, analysis of intestinal contents showed that colonization levels of CH1476-Δ*clpV* were significantly lower all along the intestinal tract than those of the CH1476 parental strain (Supplementary Fig. S4).

### Influence of T6SS-1 on the richness and diversity of intestinal microbiota in a streptomycin-treated mice model

**Figure 4:**
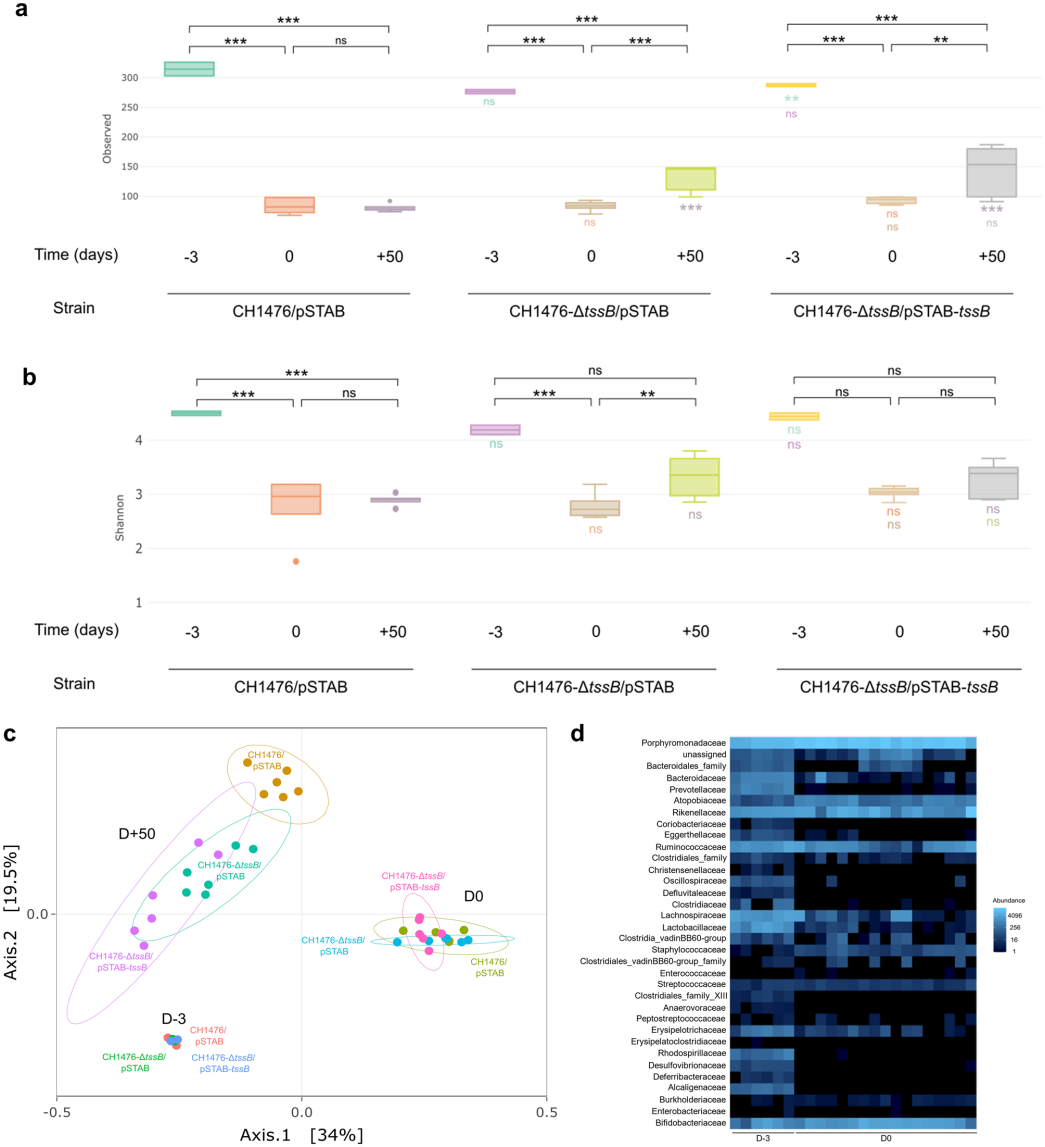
Modulation of gut microbiome of *K. pneumoniae*-infected mice correlated with the presence of T6SS-1. Alpha diversity richness: Observed index **(a)** and Shannon index **(b)** based on 16S rRNA genes from CH1476 (CH1476/pSTAB), T6SS-1 derived mutant (CH1476-Δ*tssB*/pSTAB) and T6SS-1 derived mutant trans-complemented (CH1476-Δ*tssB*/pSTAB-*tssB*) infected mice. Data are expressed as median ± minimum and maximum values. **(c)** Principal-coordinate analysis plot (PCoA) of the gut microbiota based on the results of the unweighted UniFrac non-metric multidimensional scaling (NMDS). Each point represents a mouse. **(d)** Clustering heatmap of family raw abundances in fecal microbiota of mice before (D-3) and after streptomycin treatment (D0). Blue denotes increased abundance, and black denotes decreased abundance. Statistical analysis: one-way ANOVA with post-hoc Tukey HSD test; *P < 0.05, **P < 0.01, ***P < 0.001, and ‘ns’ indicates that the difference is not significant; a color code indicates differences between mice groups at the same time point.

Overall microbiota diversity was greatly affected by initial streptomycin treatment in every animal group, as shown by the decrease in α-diversity indexes (Observed and Shannon) between D-3 and D0 (Fig. 4a and b). Principal coordinate analysis (PCoA) of unweighted UniFrac non-metric multidimensional scaling (NMDS) (β-diversity) showed that the microbial composition of mice microbiota was clearly impacted due to the streptomycin treatment (D-3 versus D0) (Fig. 4c). Some bacterial families were not affected by streptomycin administration (Porphyromonadaceae, Bifidobacteriaceae, Atopobiaceae, Rikenellaceae) while some others were significantly depleted (Bacteroidales, Prevotellaceae, Coriobacteriaceae, Oscillospiraceae, Defluvitaleaceae, Clostridiaceae, Anaerovoraceae, Rhodospirillaceae, Desulfovibrionaceae, Deferribacteraceae, Alcaligenaceae) (Fig. 4d; Supplementary Fig. S6b).

In animals receiving the *K. pneumoniae* wild-type strain (CH1476/pSTAB), the diversity of the microbiota remained low even after 50 days, whereas in animals receiving the mutant strain (CH1476-∆*tssB*/pSTAB) diversity increased (Fig. 4a and b). Diversity was only partially restored in mice receiving the trans-complemented strain (CH1476-∆*tssB*/pSTAB-*tssB*) (Fig. 4a and b). At D + 50, the β-diversity of each group of mice (receiving CH1476/pSTAB, CH1476-Δ*tssB*/pSTAB and CH1476-Δ*tssB*/pSTAB-*tssB*) was reflected in different clusters (Fig. 4c), indicating a significant effect of the cKp T6SS-1, which is likely the system responsible for delivering Tle1, on the resilience of the intestinal microbiota. Similar results were obtained with the CH1476-Δ*clpV* mutant compared to the CH1476 WT strain in in vivo assays (Supplementary Fig. S5).

**Figure 5:**
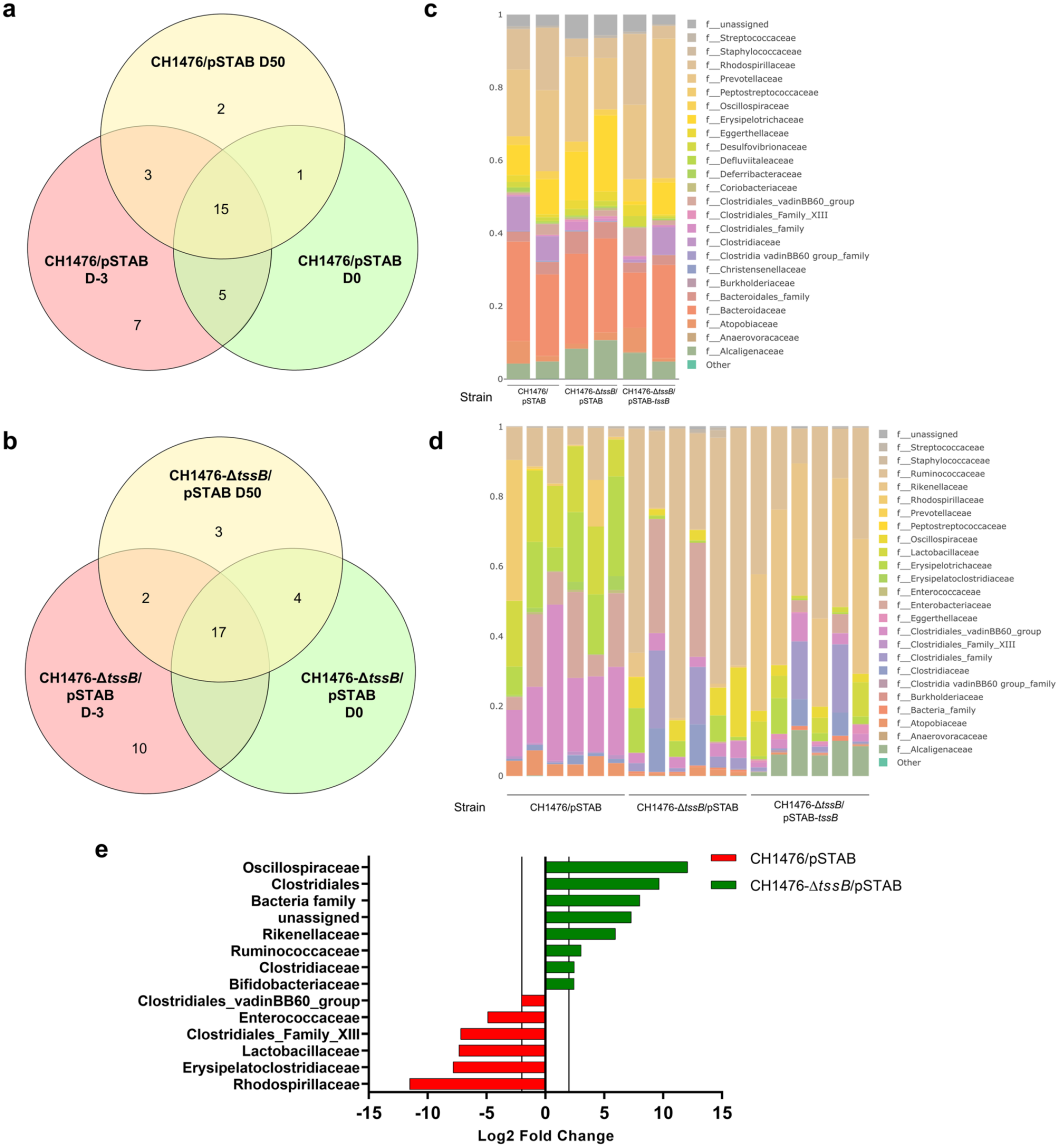
Modulation of composition of mice intestinal microbiota linked to the presence of *K. pneumoniae* and role of T6SS-1. Venn diagrams for number of bacterial families in fecal microbiota shared among the different infected groups of mice: (**a)** WT CH1476 (CH1476/pSTAB) and (**b)** T6SS-1 derived mutant (CH1476-Δ*tssB*/pSTAB) at different time points. Community composition plot for relative abundance of bacterial families in fecal microbiota at **(c).** D-3 and (**d)** D+50 infection time points in the different infected groups of mice. Each bar represents a single mouse. **(e)** Differential analysis showing families’ relative abundance between WT CH1476 (red) and T6SS-1 derived mutant (green) at D+50 PI. The dashed line indicates the limit of detection (− 2 or + 2 Log_2_ Fold Change).

Further analysis of the composition of the microbiota members in each group of mice by 16S sequencing of animal fecal content indicated that Clostridiales*,* Enterobacteriaceae and Rhodospirillaceae were present at both D-3 and D+50 in mice infected with the CH1476/pSTAB WT strain (Fig. 5a,c, and d). In the group of mice infected by CH1476-Δ*tssB*/pSTAB-*tssB*, Alcaligenaceae, Clostridiales and Anaerovoraceae families were found at both D-3 and D+50 (Fig. 5c and d; Supplementary Fig. S6a). In the group of mice infected by the CH1476-∆*tssB*/pSTAB strain, Oscillospiraceae and Alcaligenaceae families were found at D-3 and D+50 (Fig. 5b, c and d). In mice infected with the CH1476-Δ*clpV* mutant*,* Oscillospiraceae were also found at D+50 (Supplementary Fig. S7). These results suggest that Oscillospiraceae could be a potential target of T6SS of *K. pneumoniae*. Additionally, enriched and depleted families in each group were analyzed at D+50 post-infection. A 6 and 12.1-fold increase was found at D+50 in the Oscillospiraceae family in mice groups receiving, respectively, CH1476-Δ*clpV* or CH1476-*ΔtssB*/pSTAB mutant in comparison to their respective control WT group (Fig. 5e; Supplementary Fig. S7f.). Clostridiaceae was also over-represented in the two groups of mice receiving the mutant strains at D+50 in comparison with their respective WT groups (13 and 2.47-fold increase, respectively).

## Discussion

In agreement with previously published data^28,29,33,36,40^, in silico analysis detected T6SS-related genes in both hypervirulent and non-hypervirulent *K. pneumoniae* strains and in other *Klebsiella* species irrespective of the strains’ virulence and their origin of isolation. The T6SS prevalence ranged from 98% in *K. pneumoniae* species to 14% in *K. quasipneumoniae similpneumoniae*, suggesting that a strong counter selection had occurred in this subspecies, which includes mostly lower virulent strains with less extensive antibiotic resistance^41^. Two T6SS-apparented clusters, T6SS-1 and T6SS-2, were identified in the genome of the classical *K. pneumoniae* CH1157 strain. The cluster organization of T6SS-1 presents a synteny with that of T6SS-1 of the hypervirulent strain NTUH-K2044^42^. A toxin-antitoxin system was also identified in the T6SS-1 cluster composed of a type VI lipase effector 1 encoding gene (*tle1*). In accordance with the classification proposed by Li et al., a DUF2235 domain is present in the protein sequence encoded by the *tle1* gene, its specific function is not known. The presence of such a domain in CH1157 Tle1, allows its affiliation into the 4th effector group^36^. Although 80% of ST11 isolates of *K. pneumoniae* belong to this group, this is not the case for the ST2 isolate CH1157 strain. As previously mentioned, three *tli1* encoding genes, which could serve as Tle1 immunity proteins, were found upstream *tle1* in T6SS-1 cluster^36^. Tle1 phospholipase recruitment requires a DUF4123 adaptor to be fixed on the tip of the T6SS syringe before delivery. This effector chaperone is located upstream of the toxin-antitoxin system and has been found in other T6SS operons^43,44^. The presence of this potential lipase effector Tle1 was detected in 57% of *K. pneumoniae* isolates, a proportion consistent with that of previous published data obtained with Gram-negative species (*Burkholderia thailandensis*, *E. coli*, *Pseudomonas aeruginosa*, *S. enterica*, *Acinetobacter baumanii*)^34^. The CH1157 *tle1* gene has a 92% nucleotide identity coverage compared with the *tle1* sequence previously described in the multidrug-resistant *K. pneumoniae* strain HS11286^30^.

The role of T6SS in *K. pneumoniae* species has been so far studied mainly in hypervirulent strains^30–32,42^. We aimed to identify the potential antibacterial effect of *K. pneumoniae* CH1157 Tle1. As reported in another non-hypervirulent but multi-drug resistant strain, HS11286, the Tle1 effector of *K. pneumoniae* CH1157 strain could exert an antibacterial effect^30^. This toxic effect was first assessed by in vitro competition assays in i) an interspecies manner, by co-cultivating a CH1157 attacker strain and a variety of Gram-negative potential prey strains representative of those found in mice microbiota (*E. coli*, *Bacteroides thetaiotaomicron*, *Bacteroides fragilis*, *Parabacteroides dystasonis*) and ii) in an intraspecies manner, by co-cultivating a CH1157 attacker strain and its T6SS-derived mutant strains impaired either for T6SS core gene synthesis (CH1476-Δ*tssB*) or for toxin-antitoxin system production (CH1476-Δ*TA*), respectively (data not shown). No antibacterial effect was observed in the tested conditions. A different genetic background between the cKp CH1157 and the MDR HS11286 strains could also explain the difference in the in vitro effect of Tle1. In vivo, different stressors could induce expression of T6SS and/or facilitate Tle1 activity as was observed for other Enterobacteriaceae^45^. Moreover, to understand the role of this effector in a heterologous system, the *tle1* gene was cloned into pBAD33 plasmids to directly produce it either in periplasmic or in cytoplasmic compartments of *E. coli* DH5α cells. Tle1 periplasmic production in *E. coli* DH5α strain did not induce cell lysis but impaired membrane permeability as measured by IP staining, and gave rise to a small colony phenotype. Similarly, Liu et al. demonstrated the antibacterial effect of HS11286 Tle1 phospholipase in an analogous system of periplasmic production of this effector in BL21 *E. coli* cells^30^. T6SS expression in the carbapenem-resistant HS11286 strain is upregulated in the presence of β-lactam antibiotics^30^. The authors performed *E. coli* toxicity assays in presence of 50 µg/mL of ampicillin, which upregulates T6SS expression and could, in synergy with Tle1, enhance membrane permeability and thereby lead to efficient cell toxicity. The T6SS-2 cluster found in CH1157 slightly differ from the T6SS-1 since it does not encode essential T6SS core components (TssBC, Hcp). As investigated by Storey et al., there is a great diversity in T6SS cluster organization in *K. pneumoniae* genomes^46^. Other *K. pneumoniae* genomes also possess incomplete T6SS clusters, whose role remains to be defined^40^. The independent functioning of truncated T6SS clusters is still questionable: their activity could be dependent of the simultaneous presence of a complete cluster as already described for orphan genes^38^*.* The CH1157 T6SS-2 possesses an endopeptidase-encoding gene (Fig. 1c) and its putative protein (Tke) contains a N-terminal LysM peptidoglycan binding domain and a M23 putative catalytic site in the C-terminal part. No cognate immune protein was identified around the Tke potential effector, likely due to the lack of data in the immune protein database^36^. Bioinformatic analyses have shown that CH1157 Tke are mainly found in *K. pneumoniae* species and share specific domains of other T6SSs effectors (Type VI Amidase Effector (Tae) and Type VI Metallopeptidase Effector (Tpe))^47^. Moreover, an SLT transglycosylase domain (PF 01464.23) previously identified in Tke was shown to belong to Lyz-like superfamily (cl00222) and to share domains with 11th effector group in *K. pneumoniae* described by Li et al.^36^. T6SS targeted cells could therefore be affected in their capacity to survive in a competitive and stressful environment such as the intestinal tract.

Our results showed that T6SS-1 facilitates long-term establishment of *K. pneumoniae* CH1476 in the gastro-intestinal tract of animals previously treated with streptomycin. Analyses of microbiota α-diversity of mice feces harvested both pre- and post-treatment (DO versus D-3) show that streptomycin affects microbiota richness, with a decrease in facultative anaerobes (especially *Bacteroides* genus) (Fig. 4d) as previously reported^48^. The results obtained with this animal model correlate with the long-term gastrointestinal carriage of *K. pneumoniae* in patients treated with antibiotics, indicating that intestinal microbiota dysbiosis promotes *K. pneumoniae* implantation^7^. T6SS has been previously shown to contribute to the overall fitness of hvKp in vivo, and its importance in gastrointestinal colonization and translocation was reported in a murine model with strain NTUH-K2044 a short time point after challenge (2 and 6 days post-infection)^42^. Lethal effects associated with the expression of T6SS were also reported with the hvKp 52.145 strain in a *G. mellonela* larvae infection model^46^ and a murine pulmonary infection model^29^. In the latter study, trans-complementation of the isogenic Δ*pld1* mutant, deficient for the synthesis of the phospholipase effector, only partially restored the lethality phenotype. Similarly, trans-complementation of the *tssB* gene in our study partially restored the lack of colonization in mice, probably due to stoichiometry modifications of the molecular compounds necessary for T6SS machinery establishment.

T6SS-1 is necessary for the colonization of the uppermost part of the intestinal tract of mice i.e. the proximal intestine and ileal loop. Major differences in microbiota composition along the mice gastrointestinal tract could explain the disparities in colonization linked to the presence of potential *K. pneumoniae* competitors: Lactobacillaceae are predominant in the oxygen-rich small intestine, and strictly anaerobic Bacteroidaceae and Lachnospiraceae are enriched in the large intestine and fecal content of mice^49^. The role of *K. pneumoniae* T6SS within the upper GIT microbiota remains to be unraveled. Colonization gradient of *K. pneumoniae* along the intestinal tract of mice is correlated with differences in concentrations of T6SS-triggering molecules, as the concentration peak of bile salts is observed in the proximal ileum and progressively decreases distally^50^. In vitro*,* the influence of intestinal parameters on T6SS expression has already been described with intestinal pathogens: the *V. cholerae* T6SS is activated by mucins and microbiota-modified bile salt^51^, the *S.* Typhimurium T6SS is activated by bile salts^26^, and the enteroaggregative *E. coli* Sci-1 T6SS is responsive to iron starvation^52^. In hvKp, other environmental factors such as presence of anti-microbial peptides (HBD-3), high osmotic pressure and pH variations influence the expression of T6SS-encoding genes^30,42,46^. More recently, overexpression of two different clusters of T6SS in *K. pneumoniae* ST258 was correlated, in vitro and in vivo, with an excess of oxidant stress such as encountered during airway infection suggesting a role of T6SS in pulmonary pathogenesis^33^.

Antibacterial activity of T6SS is involved in bacterial warfare, even in complex environments such as plants, insects, and the gut microbiota of mammals^53^. The study of interactions between commensal microbes and gastrointestinal pathogens is a growing field, and in vivo T6SS-mediated bacterial competition has already been observed in *Salmonella* enterica Typhimurium, *Burkholderia thailandensis*, *E. coli* (EHEC), *Shigella sonnei*, *Bacteroides fragilis* and *Vibrio cholerae*^54^. However, it is clearly advised that long-term experiments should understand the real contribution of antibacterial weapons in shaping microbial communities^54^. In our study, we showed that T6SS-1 was required for intestinal persistence of cKp, suggesting that this system mediates antagonistic bacterial interactions that impair microbiota resilience. Indeed, at day 50 after infection, when the WT strain and T6SS-1 mutant were still present in equal amounts in the feces of mice, the composition of microbiota in the different groups of infection was heterogeneous. There was a higher resilience of microbiota in the CH1476-∆*tssB*/pSTAB group of co-housing mice than in the two other groups, suggesting that T6SS affects the ability of the microbiota to refill itself after breakdown. The Oscillospiraceae family was significantly increased in absence of *K. pneumoniae* T6SS-1 and could thus be a potential target of T6SS since this family is present in the intestinal microbiota of mice infected with CH1476-∆*tssB*/pSTAB at both D-3 and D+50 (Fig. 5b). As previously suggested, T6SS effectors could mediate the specific elimination of metabolic competitors^54^, thus facilitating the colonization process. For instance, *Vibrio fischeri* specifically targets competitors via TasL lipoprotein cell–cell attachment^55^. An impact of T6SS-1 effectors on eukaryotic cells cannot be totally excluded, although no Tle1 direct effect on host cells has yet been reported. However, in *P. aeruginosa*, other Tle phospholipases (PldA / Tle5A, PldB / Tle5B and TplE / Tle4) have been shown as capable of targeting eukaryotic cells (activating PI3K/Akt pathway and promoting autophagy, respectively)^21,35^.

Our approach allowed a better understanding of bacterial interactions in gut microbiota mediated by the T6SS-1 of cKp. There is a clear knowledge gap in understanding the T6SS function in the context of the holobiont and more research in this area is necessary, especially considering the diversity of T6SS effectors and the activities that may have. Indeed, the effect on other microbiota members (fungi) and host cells^21^ remains to be investigated^46^. T6SS eukaryotic-targeting effectors could enhance pathogen virulence by modulating host epithelial cell integrity or protecting themselves from innate immune response^56^. Overcoming colonization resistance by T6SS related to modulation of host response during infection, making T6SS an efficient virulence factor for pathogens^25^. Improved mechanistic understanding of commensal–pathogen interactions is a fundamental prerequisite for the design of prophylactic and therapeutic approaches for *Klebsiella* control, especially for multidrug resistant strains.

## Materials and methods

### Identification of T6SS clusters in *Klebsiella* genus

The list of genomes used in this study is presented in Supplementary Table S1a. (i) KL427 database. All fully sequenced *Klebsiella* genomes from NCBI RefSeq were downloaded and analyzed (ftp://ftp.ncbi.nih.gov/genomes/, accessed in April 2019). Species assignation was corrected using Kleborate and virulence score assignation according to the presence of well-described virulence factors^57^. The species was assigned with a strong level of confidence for 414 genomes, and considered weak for 10 (Supplementary Fig. S2a). Three strains were discarded due to wrongful genus assignation. Additional *K. quasipneumoniae subsp similpneumoniae* genomes were downloaded from NCBI on July 6th 2022 (Supplementary Table S1b), and verified with Kleborate for correct species assignation. (ii) KL65 database. This database reflects *Klebsiella* diversity in terms of species and STs and was manually curated based on the availability of reliable isolation data.

### Identification of T6SS systems

MacSyFinder^37^, an open-source software that detects macromolecular systems, was used to identify T6SS systems in *Klebsiella* genomes. As input, it requires: (i) a proteome, (ii) a set of Hidden Markov Model (HMM) protein profiles, and (iii) a model describing the number of genetic components and their organization. (i) *Definition of HMM profiles*. The HMM protein profiles have been described elsewhere^38^. Profiles for TssA, TssB, TssC, TssD (Hcp), TssE, TssF, TssG, TssH (ClpV), TssI (VgrG), TssJ, TssK, TssL, TssM and EvpJ, were used. (ii)* Model.* To identify *bona fide* T6SS, in *Klebsiella* genomes, the model corresponding to T6SSi was applied^38^, which sequentially requires 3 to 13 minimum mandatory proteins (biologically essential components for a putative functional system), associated with the T6SS. The different cluster organizations as well as their respective prevalence are presented in Supplementary Fig. S1. The different models gave qualitatively very similar results (Supplementary Fig. S2b). Decreasing the number of proteins required for a system to be considered complete resulted in an increase in secondary T6SS loci but had little effect on the number of genomes coding at least one T6SS (Supplementary Fig. S2c). The presence of at least 8 different core proteins was considered for the estimation of the prevalence of T6SS clusters, as it is the greatest number of necessary core proteins required to detect at least 2 T6SS in *Klebsiella* strains without inducing false negative results (Supplementary Figs. S2b and c). Additionally, components should be encoded in a single locus and the maximum distance between consecutive elements limited to 20 proteins. Models were written in plain text using a specific XML grammar and can be modified by the user (see http://macsyfinder.readthedocs.io/en/latest/ for details). (iii)* Identification of effectors.* BLASTP (v BLAST 2.7.1+, default parameters) were performed to identify homologous Tle1-like proteins in genomes of KL65 database to Tle1 protein encoded in the first T6SS locus of CH1157. At least 80% of protein identity was required to consider Tle1-like presence. Similarly, the presence of Tke-like proteins was evaluated by BLASTP (v BLAST 2.7.1+, default parameters) using the sequence of the endopeptidase of CH1157 as query against the RefSeq database (April 2019). The latter comprises a total of 14,363 chromosomes and 11,806 plasmids, representing 13,514 complete genomes from 4232 bacterial species. At least 50% identity and a coverage of over 60% of the protein length (> 400) was required to consider Tke-like presence.

### *Klebsiella* phylogeny (KL65 database)

(i) *Identification of the persistent genome.* To build the phylogeny, we first identified the persistent genome using PanACoTA^58^. The pan genome was inferred with the connected-component clustering algorithm of MMSeqs2 with pairwise bidirectional coverage > 0.8 and sequence identity > 0.8. The persistent genome was built from the pangenome, with a persistence threshold of 95%, meaning that a gene family had to be present in a single copy in at least 62 out of the 65 genomes to be considered persistent. Of the 22,288 gene families of the *Klebsiella* pangenome, 2969 were present in 95% of the genomes.

(ii) *Construction of the phylogenetic tree*. To compute the phylogenetic tree, families of the persistent genome were individually aligned with mafft (v7.407) (*PMID: 23329690*) from the protein sequences back-translated to DNA. The maximum likelihood tree was built using the nucleotide alignment of the persistent genome with IQ-TREE multicore (v2.1.2) under the GTR model with a total of 651,381 informative sites^59^. Information on strain collection regarding strains isolated from the hospital, community-acquired infections, the environment or carriage strains was reliable.

### Bacterial strains, plasmids and culture conditions

Bacterial strains and plasmids used in this study are listed in Table 1. CH1476 corresponds to CH1157 strain deleted for ampicillin *shv-1* B-lactamase resistance gene and replaced by *aadA7* gene conferring streptomycin resistance to allow molecular biology and in vivo experiments^39^. Derived mutant strains (T6SS mutants) were constructed as described elsewhere using the Datsenko & Wanner method^60^. Unless otherwise indicated, bacteria were grown in lysogeny broth (LB) medium in glass tubes at 37 °C in a shaker (200 rpm), or on LB agar plates overnight at 37 °C. When required, antibiotics in media were added at the following concentrations: streptomycin (50 µg/mL), ampicillin (100 µg/mL), kanamycin (50 µg/mL), tetracycline (35 µg/mL), spectinomycin (50 µg/mL), chloramphenicol (35 µg/mL).

**Table 1:** Bacterial strains and plasmids used in this study.

Identification	Description	Source and/or reference
**Bacterial strains**		
*K. pneumoniae* CH1157	*K. pneumoniae* clinical isolate, Ap^R^	^39^
*K. pneumoniae* CH1476	CH1157-Δ*shv1*::*aadA7*, Sp^R^	^39^
*K. pneumoniae* CH1476-Δ*clpV*	*clpV* mutant strain after Km cassette excision, Sp^R^	This study
*K. pneumoniae* CH1476-Δ*tssB*	*tssB* mutant strain after Km cassette excision, Sp^R^	This study
*E. coli* DH5α	F-, Δ(*argF-lac*) U169, *phoA*, *supE44*, Δ(lacZ)M15, *relA*, *endA*, *thi*, *hsdR*	Laboratory collection
**Plasmids**		
pKOBEG199	pBAD cloning vector harboring λphage red*γβα* operon, Cm^R^	^65^
pKD4	Plasmid with FRT-flanked Km-resistance cassette used for Km cassette amplification, Ap^R^, Km^R^	^60^
pCP20	Plasmid carrying the yeast recombinase gene (FLP, aka exo), Cm and Ap resistant gene and temperature sensitive replication, Ap^R^	^60^
pSTAB	pZE derivative plasmid. Contains the flm toxin-antitoxin system from F plasmid, Ap^R^	^61^
pSTAB-*tssB*	pSTAB carrying a copy of *tssB* gene, Ap^R^	This study
pBAD33	Plasmid for cloning, L-arabinose inducible, p15A origin, Cm^R^	^66^
pBAD33-ssOmpA	pBAD33 with signal peptide periplasmic specific Outer Membrane Protein A sequence inserted before MCS, Cm^R^	Gift from E. Cascales
pBAD33-ssOmpA-*tle1*	pBAD33-ssOmpA carrying a copy of *tle1* gene, Cm^R^	This study
pBAD33-RBS	pBAD33 with ribosome-binding site consensus sequence inserted into MCS, Cm^R^	^66^
pBAD33-RBS-*tle1*	pBAD33-RBS carrying a copy of *tle1* gene, Cm^R^	This study

*Abbreviations* Ampicillin (Ap); Spectinomycin (Sp); Kanamycin (Km); Tetracycline (Tet); Chloramphenicol (Cm).

For trans-complementation assays, DNA fragments containing the entire *tssB* gene were amplified from *K. pneumoniae* CH1476 genomic DNA by an overlapping extension PCR using primers listed in Supplementary Table S2 (Ins-*tssB*-pSTAB-Fw and Ins-*tssB*-pSTAB-Rv). The resulting fragment was cloned into the linearized stable plasmid pSTAB^61^ previously amplified with the primers Lin-pSTAB-*tssB*-Fw and Lin-pSTAB-*tssB*-Rv (Supplementary Table S2) using Gibson Assembly Cloning Kit (NEW ENGLAND BIOLABS; Ipswich, Massachusetts, USA).

Plasmid vectors pBAD33-ssOmpA (periplasmic addressing of the product) and pBAD33-RBS (cytoplasmic addressing of the product) (Table 1) were used in cloning experiments^62^ performed in the *Escherichia coli* DH5α strain. The *tle1* gene was amplified with high fidelity Phusion Taq (THERMOFISHER; Waltham, Massachusetts, USA) from CH1476 genomic DNA using oligonucleotides (Supplementary Table S2) containing *SalI* and *HindIII* restriction sites within their 5′ and 3′ ends respectively. The resulting *tle1* amplification product was digested by *SalI* and *HindIII* restriction enzymes and then inserted into both pBAD33 plasmids (pBAD33-ssOmpA-*tle1* and pBAD33-RBS-*tle1*) (Table 1). After electroporation, transformants were selected onto LB agar plates containing 0.8% glucose to inhibit “leaky expression” of the pBAD33 promoter and 35 µg/mL chloramphenicol. All constructions were verified by PCR (GoTaq Colorless Mastermix) (PROMEGA; Charbonnières les Bains, France) and by determination of DNA sequence (EUROFINS GENOMICS; Ebersberg, Germany).

### Effect of *tle1* expression in *E. coli* DH5α

Measurement of the Tle1 effect was assessed in *E. coli* DH5α pBAD33-derived strains (**Table ****1**) by (i) bacterial growth monitoring and CFU counts, (ii) phenotypical analysis by agar plates spotting, and (iii) flow cytometry analysis after live-dead staining. (i) Briefly, overnight cultures in LB supplemented with 0.8% glucose were diluted to an OD_620nm_ 0.001 in LB medium containing 0.2% arabinose and their growth was monitored for 8 h by OD_620nm_ measurements. At the last time point, serial dilutions of the suspensions were plated onto LB agar supplemented with 0.8% glucose and chloramphenicol for CFU counting; (ii) O/N cultures adjusted to an OD_620nm_ 0.2 were serially diluted, and 10µL of each dilution were spotted onto LB agar containing 0.2% arabinose and chloramphenicol; (iii) Cells from 8-h culture were harvested by centrifugation from batch culture (at 3800*G*), washed once with saline solution, and diluted 50-fold prior to live/dead staining. A standard nucleic staining protocol combining the cell-permeant fluorochrome SYBR Green I (SGI, THERMOFISHER) and the cell-impermeant Propidium Iodide (PI, SIGMA-ALDRICH; St-Quentin-Fallavier, France) was used to reveal live and dead cells. SG I (10,000 × stock) was added to the sample (1× final concentration) and incubated for 10 min before the addition of PI (10 µg/mL fc) for an additional 5 min, in the dark and at room temperature. Tubes containing stained cells were vortexed for 10 s before acquisition (flow rate 69.2μL/min, and at least 20,000 events collected) with a BD LSR Fortessa X20 flow cytometer (BD BIOSCIENCES; Le Pont de Claix, France). SGI and PI were excited by a 488 nm (50mW) laser and collected through a 530/30BP filter with a preceding 505LP filter for SGI and through a 610/20BP filter with a preceding 600LP filter for PI. Counts were obtained in a bivariate dot plot in which live and dead cell populations were divided according to SYBR emission (on the abscissa axis) and PI emission (on the ordinate axis).

### In vivo gastrointestinal colonization assays

Eight-week-old specific pathogen-free C57BL-6 J male mice (CHARLES RIVER; Saint Germain Nuelles, France) were housed six per cage at animal biosafety level 2 (21–22 °C, 12 h/12 h light–dark cycle) with access to food and water ad libitum. All experiments were performed in compliance with ARRIVE guidelines and according to the ethical guidelines set out in the Guide for the Care and Use of Laboratory Animals and with approval of the “Comité d’Ethique pour l’Expérimentation Animale Auvergne” (C2E2A), the local ethics committee (Reference number: APAFIS#17520-2019030413278293). After + /− pre-treatment for 72 h with 5 g/L of streptomycin in drinking water, mice received 1 × 10^8^ CFU equivalent doses of *K. pneumoniae* WT and derived strains in 200µL of PBS via the intragastric route. Feces were first collected daily, then weekly, weighed, and homogenized in PBS (500–1000 µL). Serial dilutions were plated onto selective LB agar media with an appropriate antibiotic (ampicillin 100 µg/mL) to evaluate *K. pneumoniae* colonization levels in mouse intestine (Fig. 3a). Results were expressed as Log_10_ of numbers of CFU/g of feces. Before antibiotic treatment (D-3), the day of infection (D0) and 50 days after inoculation (D50), feces were also collected and frozen at − 80 °C until nucleic acid extraction. At the end of the experiment, mice were killed by cervical dislocation in strict accordance with ethical recommendations. The contents of different gastrointestinal tract parts (proximal, ileal loop, caecum, colon) were collected, serially diluted in PBS and plated onto selective LB agar with an appropriate antibiotic (ampicillin 100 µg/mL) to assess pathogen distribution throughout the gastrointestinal tract. Results were expressed as Log_10_ of numbers of CFU/mg of content.

### DNA extraction from feces and 16S sequencing

DNA was extracted from mice feces with the FastDNA Spin Kit for Soil (MPBIOMEDICALS) according to the manufacturer’s instruction**s**. Total nucleic acid extracts were eluted in 50µL of DES Buffer. DNA content of each sample was assessed with Qubit (THERMOFISHER). Samples were stocked at − 20 °C until 16S DNA sequencing. V3 and V4 regions of 16S rRNA gene were amplified using PCR1F_460-PCR1R_460 primer pair (Supplementary Table S2). The amplicons were sequenced using Illumina Miseq (ILLUMINA INC; Waltham, San Diego, CA, USA) at the INRAE GeT platform (GenoToul, Castanet-Tolosan, France), generating 250 bp paired-end reads. Raw data were processed with the rANOMALY^63^ which relies on the dada2 R package to produce amplicon sequence variants (ASV) as taxonomic units. The resulting ASV table was filtered with a minimum relative abundance of 5 × 10^–5^ and used to perform statistical and diversity analyses.

### Statistical analysis and data representation

Statistical analyses were performed with GraphPad Prism 9 (GraphPad Software; La Jolla, CA, USA). Amplicon sequencing data were explored with R version 3.5.3 and Explore Metabar, accessible from the Migale server (https://shiny.migale.inrae.fr/app/exploremetabar)^63^. Alpha diversity was explored with the Observed index for richness and the Shannon index for evenness. Pairwise comparison between time points were tested with TukeyHSD test and adjusted *P*-values were reported. Distances between samples (Beta diversity) were measured with unweighted unifrac distances and projected on a two dimensional spaces by Non-metric Multidimensional Scaling (NMDS). Group of samples are compared by a pairwise Adonis test. A *P*-value of less than 0.05 was considered as significant.

## Supplementary Information

Supplementary Information 1.

Supplementary Information 2.

Supplementary Information 3.

## Data Availability

The 16S rRNA gene sequences datasets generated and analyzed in this study are available in the NCBI repository with the BioProject ID: PRJEB52868 (https://www.ncbi.nlm.nih.gov/).
