# Supplementary material for: Role of Klebsiella pneumoniae Type VI secretion system (T6SS) in long-term gastrointestinal colonization
Source: Sci Rep. 2022 Oct 10;12:16968. doi: 10.1038/s41598-022-21396-w (PMC9550808; doi:10.1038/s41598-022-21396-w)
Supplement: Supplementary file 1 — Supplementary Information 1. [file 41598_2022_21396_MOESM1_ESM.pdf]

<u>Title:</u> **Role of *Klebsiella pneumoniae* Type VI secretion system (T6SS) in long-term gastrointestinal colonization**

Thomas MERCIECCA[1], Stéphanie BORNES[2], Laurence NAKUSI[1], Sébastien THEIL[2], Olaya RENDUELES[3], Christiane FORESTIER[1], Sylvie MIQUEL[1*]

[1] Université Clermont Auvergne, CNRS, LMGE, F-63000 Clermont–Ferrand, France.
[2] Université Clermont Auvergne, INRAE, VetAgro Sup, UMRF, F-63000 Clermont–Ferrand, France
[3] Institut Pasteur, Université de Paris, CNRS, UMR3525, Microbial Evolutionary Genomics, F-75015, Paris, France

[*]Corresponding author: Sylvie Miquel (sylvie.miquel@uca.fr)

Supplemental Content Legends

Table S1a. KL247 genomes database.

Table S1b. *K. qs. similipneumoniae* genomes database.

Table S2. Primers used in this study.

Figure S1. T6SS clusters organization and frequency. Frequency of the given organization considering at least 8 proteins between TssA, TssB, TssC, TssD (Hcp), TssE, TssF, TssG, TssH (ClpV), TssI (VgrG), TssJ, TssK, TssL, TssM and EvpJ (PAAR). The number next to TssX protein is the number of each protein present in this particular organization.

Figure S2. Characterization of the *Klebsiella* database and T6SS. a. Robustness of the species classification of each genome, expressed as confidence level by Kleborate. **b.** Frequency of the number of T6SS systems per genome detected by the model when applying different thresholds in the minimum mandatory proteins required to detect a system as complete. This

ranged from 3 to 13. **c.** Number of genomes with complete T6SS depending on the number of minimum mandatory proteins required to detect a system as complete.

Figure S3. Identification of metallopeptidase in bacterial genomes. a. A BlastP against the RefSeq database, accessed in April 2019 and comprising a total of 14363 chromosomes and 11806 plasmids, and representing 13514 complete genomes from 4232 different bacterial species, was performed with default parameters using the metallopeptidase of CH1157 as query. The percentage of identity, the length of the alignment and the log10-transformed e-value are indicated for all hits. **b**. Number of metallopeptidases identified with an identity percentage of more than 50 %, and a coverage of over 60 % of the protein length (length > 400). Numbers on top of bars correspond to the percentage of prevalence across the genomes of the species. Empty bars indicate species for which only one genome was analysed.

Figure S4. Role of T6SS-1 in gastrointestinal colonization of mice by *K. pneumoniae* CH1476 (CH1476-Δ*clpV* second *in vivo* assay). a. Experimental procedure (n=6/group) **b.** After 72h of streptomycin treatment (5g/L in drinking water), mice were intragastrically inoculated with $1x10^8$ CFU of *K. pneumoniae* CH1476 strain (CH1476, full circle) or the CH1476-Δ*clpV* isogenic mutant (CH1476-Δ*clpV*, full square). Results are expressed as means *K. pneumoniae* CFU/g of feces ± SEM over 110 days; the dashed line indicates the limit of detection (10^2 CFU). **c.** Representation along the gastrointestinal tract of *K. pneumoniae* CH1476 WT (black bar) or the CH1476-Δ*clpV* isogenic mutant (full line bar) in CFU/mg of content in mice. Data are expressed as means of each six values ± SEM. Statistical analysis: non-parametric Mann Whitney test; *p<0.05, **p<0.01.

Figure S5. Modulation of gut microbiome of *K. pneumoniae* infected mice correlated with the presence of T6SS-1 (CH1476-Δ*clpV* second *in vivo* assay). Alpha diversity richness:

Observed index **(a)** and Shannon index **(b)** based on 16S rRNA genes from CH1476 (CH1476) and T6SS-1 derived mutant (CH1476-Δ*clpV*) and infected mice. Data are expressed as median ± minimum and maximum values. **c.** Principal-coordinate analysis plot (PCoA) of the gut microbiota based on the results of the unweighted UniFrac non-metric multidimensional scaling (NMDS). Each point represents a mouse. **d.** Clustering heatmap of family raw abundances in fecal microbiota of mice before (D-3) and after streptomycin treatment (D0). Blue denotes increased expression, and black denotes decreased expression. Statistical analysis: one-way ANOVA with post-hoc Tukey HSD test; *p<0.05, **p<0.01, ***p<0.001, and 'ns' indicates that the difference is not significant; a color code indicates differences between mice group at the same time point.

Figure S6. Modulation of composition of mice intestinal microbiota linked to the presence of *K. pneumoniae* and role of T6SS-1 (Trans-complemented and D-3). a. Venn diagram for number of bacterial families in fecal microbiota shared at different time points in the group of mice infected by the T6SS-1 derived mutant trans-complemented (CH1476-Δ*tssB*/pSTAB-*tssB*). **b.** Community composition plot for relative abundance of bacterial families in fecal microbiota at D0 infection time point in the different infected groups of mice. Each bar represents a single mouse.

Figure S7. Modulation of composition of mice intestinal microbiota linked to the presence of *K. pneumoniae* and role of T6SS-1 (CH1476-Δ*clpV* second *in vivo* assay). Venn diagrams for number of bacterial families in fecal microbiota shared among the different infected groups of mice: **a.** WT CH1476 (CH1476) and **b**. T6SS-1 derived mutant (CH1476-Δ*clpV*) at different time points. Community composition plot for relative abundance of bacterial families in fecal microbiota at **c.** D-3, **d.** D0 and **e.** D+50 infection time points in the different infected groups

of mice. Each bar represents a single mouse. **f.** Differential analysis showing families' relative abundance between WT CH1476 (red) and T6SS-1 derived mutant (CH1476-Δ*clpV*) (green) at D+50 PI. The dashed line indicates the limit of detection (-2 or +2 Log_2 Fold Change).

Table S2. Primers used in this study

Primer name	Oligonucleotidic sequence (5' to 3')	PCR Product size (bp)	Use
clpV-FRT-Fw<br><br>*clpV-FRT-Rv*	GTTCCTGTTCAGGAACAGCGACGGGCGGTAGCGTGTCCGGAAACA ATAAGAGAGGATCTCGTCTTGAGCGATTGTGTAGG<br>GCTTCACATCCCTTCACCGAAAGCAGGTGGTGGTTATGGCTGAACA ACAACGATTTCACGTGGTCCATATGAATATCCTC	1615	Inactivation fragment synthesis for CH1476-Δ*clpV* mutant construction
tssB-FRT-Fw<br><br>*tssB-FRT-Rv*	CATCGTCACGTTTCACATCAATATATCTTCAATAATTATTCCTTAAGG AAAGGAACTGCTGTCTTGAGCGATTGTGTAGG<br>TTCTGTGGTAACAGACATTAGCATTTTCCCGGTTAATCCATTAACAG ACTTGCCCCGCACTGGTCCATATGAATATCCTCC	1615	Inactivation fragment synthesis for CH1476-Δ*tssB* mutant construction
clpV-int-Fw<br>*clpV-int-Rv*	GATGAGGTGGAAAAGGCCCA<br>AAAGTCGATTTCACGCCCCT	99	Correct insertion of kanamycin cassette for CH1476-Δ*clpV* mutant construction
clpV-ext-Fw<br>*clpV-ext-Rv*	CTCCGATAGCTGGAACGAAC<br>TCCTGGTAAATGGCGTTCTG	WT : 3325<br>Δ*clpV*: 2165	Correct insertion of kanamycin cassette for CH1476-Δ*clpV* mutant construction
tssB-int-Fw<br>*tssB-int-Rv*	AACCTCTCGGTGCCGAATAC<br>CAACCTGCTCCGGTTCAAAA	97	Correct insertion of kanamycin cassette for CH1476-Δ*tssB* mutant construction
tssB-ext-Fw<br>*tssB-ext-Rv*	ATTGCCCCGGTCGTCTCTAT<br>CCATAAACACCTGCATCGCC	WT : 1044<br>Δ*tssB*: 2047	Correct insertion of kanamycin cassette for CH1476-Δ*tssB* mutant construction
Ins-tssB-pSTAB-Fw<br>*Ins-tssB-pSTAB-Rv*	CAGGACGCACTGACCGAATTCTTTCAAATATTGATATTCAT<br>CCTTGCTGGCATTGTACGGGGGTACCTTATTTCGGGGCAAGCGCAC	710	Fragment synthesis for CH1476-Δ*tssB* + pSTAB-*tssB* transcomplemented mutant construction
Lin-pSTAB-tssB-Fw<br>*Lin-pSTAB-tssB-Rv*	GGTACCCCCGTACAATGCCA<br>GAATTCGGTCAGTGCGTCCTG	2769	pSTAB linearization for CH1476-ΔtssB + pSTAB-*tssB* transcomplemented mutant construction
Ins-Tle1-SalI-Fw<br>*Ins-Tle1-HindIII-Rv*	ATCGTGCGTCGACATGTCCGAAATAACCGAAAC<br>CGTGCTAAAGCTTTCAGACCGTTGTCGTCAGAA	2478	*tle1* gene amplification with flanked *SalI* and *HindIII* restriction site
Verif-pBAD33ssOmpA-Fw<br>*Verif-pBAD33ssOmpA-Rv*	AGTGTCTATAATCACGGCAG<br>GTCAGGTGGGACCACCGCGC	pBAD33-ssOmpA : 408<br>pBAD33-RBS : 344<br>pBAD33-ssOmpA-*tle1* : 2844<br>pBAD33-RBS-*tle1*: 2781	Correct insertion of *tle1* gene in pBAD33 vectors for directional cloning
PCR1F_460<br>*PCR1R_460*	TTACCAGGGTATCTAATCCT<br>TACGGRAGGCAGCAG	460	Verification of DNA extract for 16S sequencing

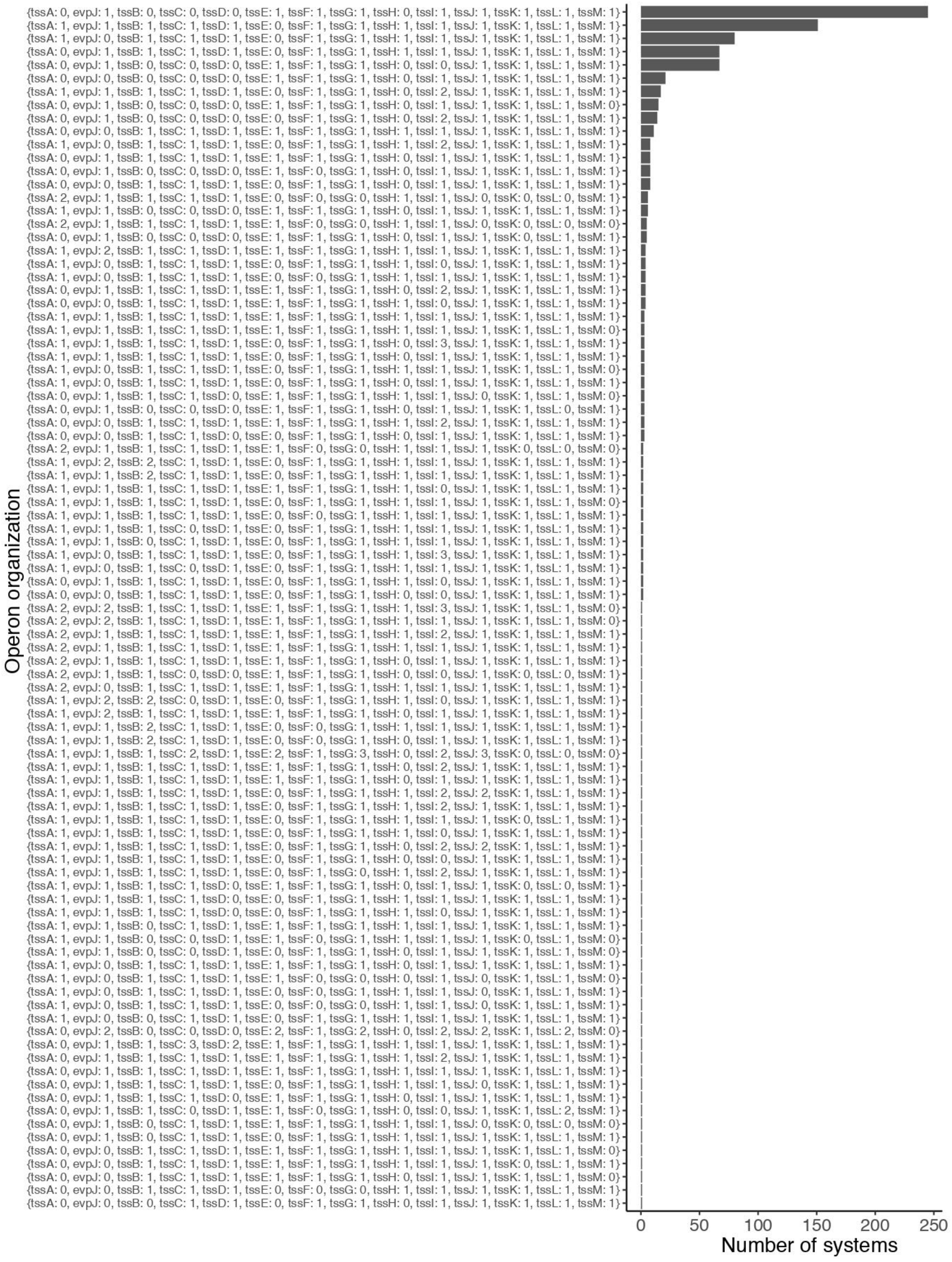
{tssA: 0, evpJ: 1, tssB: 0, tssC: 0, tssD: 0, tssE: 1, tssF: 1, tssG: 1, tssH: 0, tssI: 1, tssJ: 1, tssK: 1, tssL: 1, tssM: 1}
{tssA: 1, evpJ: 1, tssB: 1, tssC: 1, tssD: 1, tssE: 0, tssF: 1, tssG: 1, tssH: 1, tssI: 1, tssJ: 1, tssK: 1, tssL: 1, tssM: 1}
{tssA: 1, evpJ: 0, tssB: 1, tssC: 1, tssD: 1, tssE: 0, tssF: 1, tssG: 1, tssH: 1, tssI: 1, tssJ: 1, tssK: 1, tssL: 1, tssM: 1}
{tssA: 0, evpJ: 1, tssB: 1, tssC: 1, tssD: 1, tssE: 0, tssF: 1, tssG: 1, tssH: 1, tssI: 1, tssJ: 1, tssK: 1, tssL: 1, tssM: 1}
{tssA: 0, evpJ: 1, tssB: 0, tssC: 0, tssD: 0, tssE: 1, tssF: 1, tssG: 1, tssH: 0, tssI: 0, tssJ: 1, tssK: 1, tssL: 1, tssM: 1}
{tssA: 0, evpJ: 0, tssB: 0, tssC: 0, tssD: 0, tssE: 1, tssF: 1, tssG: 1, tssH: 0, tssI: 1, tssJ: 1, tssK: 1, tssL: 1, tssM: 1}
{tssA: 1, evpJ: 1, tssB: 1, tssC: 1, tssD: 1, tssE: 0, tssF: 1, tssG: 1, tssH: 0, tssI: 2, tssJ: 1, tssK: 1, tssL: 1, tssM: 1}
{tssA: 0, evpJ: 1, tssB: 0, tssC: 0, tssD: 0, tssE: 1, tssF: 1, tssG: 1, tssH: 0, tssI: 1, tssJ: 1, tssK: 1, tssL: 1, tssM: 0}
{tssA: 0, evpJ: 1, tssB: 0, tssC: 0, tssD: 0, tssE: 0, tssF: 1, tssG: 1, tssH: 0, tssI: 2, tssJ: 1, tssK: 1, tssL: 1, tssM: 1}
{tssA: 0, evpJ: 0, tssB: 1, tssC: 1, tssD: 1, tssE: 1, tssF: 1, tssG: 1, tssH: 1, tssI: 1, tssJ: 1, tssK: 1, tssL: 1, tssM: 1}
{tssA: 1, evpJ: 0, tssB: 1, tssC: 1, tssD: 1, tssE: 0, tssF: 1, tssG: 1, tssH: 1, tssI: 2, tssJ: 1, tssK: 1, tssL: 1, tssM: 1}
{tssA: 0, evpJ: 1, tssB: 1, tssC: 1, tssD: 1, tssE: 1, tssF: 1, tssG: 1, tssH: 0, tssI: 1, tssJ: 1, tssK: 1, tssL: 1, tssM: 1}
{tssA: 0, evpJ: 1, tssB: 0, tssC: 0, tssD: 0, tssE: 1, tssF: 0, tssG: 1, tssH: 0, tssI: 1, tssJ: 1, tssK: 1, tssL: 1, tssM: 1}
{tssA: 0, evpJ: 0, tssB: 1, tssC: 1, tssD: 1, tssE: 0, tssF: 1, tssG: 1, tssH: 0, tssI: 1, tssJ: 1, tssK: 1, tssL: 1, tssM: 1}
{tssA: 2, evpJ: 1, tssB: 1, tssC: 1, tssD: 1, tssE: 0, tssF: 0, tssG: 0, tssH: 1, tssI: 1, tssJ: 0, tssK: 0, tssL: 0, tssM: 1}
{tssA: 1, evpJ: 1, tssB: 0, tssC: 0, tssD: 0, tssE: 1, tssF: 1, tssG: 1, tssH: 0, tssI: 1, tssJ: 1, tssK: 1, tssL: 1, tssM: 1}
{tssA: 2, evpJ: 1, tssB: 1, tssC: 1, tssD: 1, tssE: 1, tssF: 0, tssG: 0, tssH: 1, tssI: 1, tssJ: 0, tssK: 0, tssL: 0, tssM: 0}
{tssA: 0, evpJ: 1, tssB: 0, tssC: 0, tssD: 0, tssE: 1, tssF: 1, tssG: 1, tssH: 0, tssI: 1, tssJ: 1, tssK: 0, tssL: 1, tssM: 1}
{tssA: 1, evpJ: 2, tssB: 1, tssC: 1, tssD: 1, tssE: 1, tssF: 1, tssG: 1, tssH: 1, tssI: 1, tssJ: 1, tssK: 1, tssL: 1, tssM: 1}
{tssA: 1, evpJ: 0, tssB: 1, tssC: 1, tssD: 1, tssE: 0, tssF: 1, tssG: 1, tssH: 1, tssI: 0, tssJ: 1, tssK: 1, tssL: 1, tssM: 1}
{tssA: 1, evpJ: 0, tssB: 1, tssC: 1, tssD: 1, tssE: 0, tssF: 0, tssG: 1, tssH: 1, tssI: 1, tssJ: 1, tssK: 1, tssL: 1, tssM: 1}
{tssA: 0, evpJ: 1, tssB: 1, tssC: 1, tssD: 1, tssE: 1, tssF: 1, tssG: 1, tssH: 0, tssI: 2, tssJ: 1, tssK: 1, tssL: 1, tssM: 1}
{tssA: 0, evpJ: 0, tssB: 1, tssC: 1, tssD: 1, tssE: 1, tssF: 1, tssG: 1, tssH: 1, tssI: 0, tssJ: 1, tssK: 1, tssL: 1, tssM: 1}
{tssA: 1, evpJ: 1, tssB: 1, tssC: 1, tssD: 1, tssE: 1, tssF: 1, tssG: 1, tssH: 1, tssI: 1, tssJ: 1, tssK: 1, tssL: 1, tssM: 1}
{tssA: 1, evpJ: 1, tssB: 1, tssC: 1, tssD: 1, tssE: 1, tssF: 1, tssG: 1, tssH: 1, tssI: 1, tssJ: 1, tssK: 1, tssL: 1, tssM: 0}
{tssA: 1, evpJ: 1, tssB: 1, tssC: 1, tssD: 1, tssE: 0, tssF: 1, tssG: 1, tssH: 0, tssI: 3, tssJ: 1, tssK: 1, tssL: 1, tssM: 1}
{tssA: 1, evpJ: 1, tssB: 1, tssC: 1, tssD: 1, tssE: 0, tssF: 1, tssG: 1, tssH: 0, tssI: 1, tssJ: 1, tssK: 1, tssL: 1, tssM: 1}
{tssA: 1, evpJ: 0, tssB: 1, tssC: 1, tssD: 1, tssE: 1, tssF: 1, tssG: 1, tssH: 1, tssI: 1, tssJ: 1, tssK: 1, tssL: 1, tssM: 0}
{tssA: 1, evpJ: 0, tssB: 1, tssC: 1, tssD: 1, tssE: 0, tssF: 1, tssG: 1, tssH: 0, tssI: 1, tssJ: 1, tssK: 1, tssL: 1, tssM: 1}
{tssA: 0, evpJ: 1, tssB: 1, tssC: 1, tssD: 0, tssE: 1, tssF: 1, tssG: 1, tssH: 1, tssI: 1, tssJ: 0, tssK: 1, tssL: 1, tssM: 0}
{tssA: 0, evpJ: 1, tssB: 0, tssC: 0, tssD: 0, tssE: 1, tssF: 1, tssG: 1, tssH: 0, tssI: 1, tssJ: 1, tssK: 1, tssL: 0, tssM: 1}
{tssA: 0, evpJ: 0, tssB: 1, tssC: 1, tssD: 1, tssE: 1, tssF: 1, tssG: 1, tssH: 1, tssI: 2, tssJ: 1, tssK: 1, tssL: 1, tssM: 1}
{tssA: 0, evpJ: 0, tssB: 1, tssC: 1, tssD: 0, tssE: 0, tssF: 1, tssG: 1, tssH: 0, tssI: 1, tssJ: 1, tssK: 1, tssL: 1, tssM: 1}
{tssA: 2, evpJ: 1, tssB: 1, tssC: 1, tssD: 1, tssE: 1, tssF: 0, tssG: 0, tssH: 1, tssI: 1, tssJ: 1, tssK: 0, tssL: 0, tssM: 0}
{tssA: 1, evpJ: 2, tssB: 2, tssC: 1, tssD: 1, tssE: 0, tssF: 1, tssG: 1, tssH: 1, tssI: 1, tssJ: 1, tssK: 1, tssL: 1, tssM: 1}
{tssA: 1, evpJ: 1, tssB: 2, tssC: 1, tssD: 1, tssE: 0, tssF: 1, tssG: 1, tssH: 1, tssI: 1, tssJ: 1, tssK: 1, tssL: 1, tssM: 1}
{tssA: 1, evpJ: 1, tssB: 1, tssC: 1, tssD: 1, tssE: 1, tssF: 1, tssG: 1, tssH: 1, tssI: 0, tssJ: 1, tssK: 1, tssL: 1, tssM: 1}
{tssA: 1, evpJ: 1, tssB: 1, tssC: 1, tssD: 1, tssE: 0, tssF: 1, tssG: 1, tssH: 1, tssI: 1, tssJ: 1, tssK: 1, tssL: 1, tssM: 0}
{tssA: 1, evpJ: 1, tssB: 1, tssC: 1, tssD: 1, tssE: 0, tssF: 0, tssG: 1, tssH: 1, tssI: 1, tssJ: 1, tssK: 1, tssL: 1, tssM: 1}
{tssA: 1, evpJ: 1, tssB: 1, tssC: 0, tssD: 1, tssE: 0, tssF: 1, tssG: 1, tssH: 1, tssI: 1, tssJ: 1, tssK: 1, tssL: 1, tssM: 1}
{tssA: 1, evpJ: 1, tssB: 0, tssC: 1, tssD: 1, tssE: 0, tssF: 1, tssG: 1, tssH: 1, tssI: 1, tssJ: 1, tssK: 1, tssL: 1, tssM: 1}
{tssA: 1, evpJ: 0, tssB: 1, tssC: 1, tssD: 1, tssE: 0, tssF: 1, tssG: 1, tssH: 1, tssI: 3, tssJ: 1, tssK: 1, tssL: 1, tssM: 1}
{tssA: 1, evpJ: 0, tssB: 1, tssC: 0, tssD: 1, tssE: 0, tssF: 1, tssG: 1, tssH: 1, tssI: 1, tssJ: 1, tssK: 1, tssL: 1, tssM: 1}
{tssA: 0, evpJ: 1, tssB: 1, tssC: 1, tssD: 1, tssE: 0, tssF: 1, tssG: 1, tssH: 1, tssI: 0, tssJ: 1, tssK: 1, tssL: 1, tssM: 1}
{tssA: 0, evpJ: 0, tssB: 1, tssC: 1, tssD: 1, tssE: 0, tssF: 1, tssG: 1, tssH: 0, tssI: 0, tssJ: 1, tssK: 1, tssL: 1, tssM: 1}
{tssA: 2, evpJ: 2, tssB: 1, tssC: 1, tssD: 1, tssE: 1, tssF: 1, tssG: 1, tssH: 1, tssI: 3, tssJ: 1, tssK: 1, tssL: 1, tssM: 0}
{tssA: 2, evpJ: 2, tssB: 1, tssC: 1, tssD: 1, tssE: 1, tssF: 1, tssG: 1, tssH: 1, tssI: 1, tssJ: 1, tssK: 1, tssL: 1, tssM: 0}
{tssA: 2, evpJ: 1, tssB: 1, tssC: 1, tssD: 1, tssE: 1, tssF: 1, tssG: 1, tssH: 1, tssI: 2, tssJ: 1, tssK: 1, tssL: 1, tssM: 1}
{tssA: 2, evpJ: 1, tssB: 1, tssC: 1, tssD: 1, tssE: 1, tssF: 1, tssG: 1, tssH: 1, tssI: 1, tssJ: 1, tssK: 1, tssL: 1, tssM: 1}
{tssA: 2, evpJ: 1, tssB: 1, tssC: 1, tssD: 1, tssE: 1, tssF: 1, tssG: 1, tssH: 0, tssI: 1, tssJ: 1, tssK: 1, tssL: 1, tssM: 1}
{tssA: 2, evpJ: 1, tssB: 1, tssC: 0, tssD: 0, tssE: 1, tssF: 1, tssG: 1, tssH: 0, tssI: 0, tssJ: 1, tssK: 0, tssL: 0, tssM: 1}
{tssA: 2, evpJ: 0, tssB: 1, tssC: 1, tssD: 1, tssE: 1, tssF: 1, tssG: 1, tssH: 1, tssI: 1, tssJ: 1, tssK: 1, tssL: 1, tssM: 1}
{tssA: 1, evpJ: 2, tssB: 2, tssC: 0, tssD: 1, tssE: 0, tssF: 1, tssG: 1, tssH: 1, tssI: 0, tssJ: 1, tssK: 1, tssL: 1, tssM: 1}
{tssA: 1, evpJ: 2, tssB: 1, tssC: 1, tssD: 1, tssE: 1, tssF: 1, tssG: 1, tssH: 0, tssI: 1, tssJ: 1, tssK: 1, tssL: 1, tssM: 1}
{tssA: 1, evpJ: 1, tssB: 2, tssC: 1, tssD: 1, tssE: 0, tssF: 0, tssG: 1, tssH: 1, tssI: 1, tssJ: 1, tssK: 1, tssL: 1, tssM: 1}
{tssA: 1, evpJ: 1, tssB: 2, tssC: 1, tssD: 1, tssE: 0, tssF: 0, tssG: 1, tssH: 0, tssI: 1, tssJ: 1, tssK: 1, tssL: 1, tssM: 1}
{tssA: 1, evpJ: 1, tssB: 1, tssC: 2, tssD: 1, tssE: 2, tssF: 1, tssG: 3, tssH: 0, tssI: 2, tssJ: 3, tssK: 0, tssL: 0, tssM: 0}
{tssA: 1, evpJ: 1, tssB: 1, tssC: 1, tssD: 1, tssE: 1, tssF: 1, tssG: 1, tssH: 0, tssI: 2, tssJ: 1, tssK: 1, tssL: 1, tssM: 1}
{tssA: 1, evpJ: 1, tssB: 1, tssC: 1, tssD: 1, tssE: 1, tssF: 1, tssG: 1, tssH: 0, tssI: 1, tssJ: 1, tssK: 1, tssL: 1, tssM: 1}
{tssA: 1, evpJ: 1, tssB: 1, tssC: 1, tssD: 1, tssE: 0, tssF: 1, tssG: 1, tssH: 1, tssI: 2, tssJ: 2, tssK: 1, tssL: 1, tssM: 1}
{tssA: 1, evpJ: 1, tssB: 1, tssC: 1, tssD: 1, tssE: 0, tssF: 1, tssG: 1, tssH: 1, tssI: 2, tssJ: 1, tssK: 1, tssL: 1, tssM: 1}
{tssA: 1, evpJ: 1, tssB: 1, tssC: 1, tssD: 1, tssE: 0, tssF: 1, tssG: 1, tssH: 1, tssI: 1, tssJ: 1, tssK: 0, tssL: 1, tssM: 1}
{tssA: 1, evpJ: 1, tssB: 1, tssC: 1, tssD: 1, tssE: 0, tssF: 1, tssG: 1, tssH: 1, tssI: 0, tssJ: 1, tssK: 1, tssL: 1, tssM: 1}
{tssA: 1, evpJ: 1, tssB: 1, tssC: 1, tssD: 1, tssE: 0, tssF: 1, tssG: 1, tssH: 0, tssI: 2, tssJ: 2, tssK: 1, tssL: 1, tssM: 1}
{tssA: 1, evpJ: 1, tssB: 1, tssC: 1, tssD: 1, tssE: 0, tssF: 1, tssG: 1, tssH: 0, tssI: 0, tssJ: 1, tssK: 1, tssL: 1, tssM: 1}
{tssA: 1, evpJ: 1, tssB: 1, tssC: 1, tssD: 1, tssE: 0, tssF: 1, tssG: 0, tssH: 1, tssI: 2, tssJ: 1, tssK: 1, tssL: 1, tssM: 1}
{tssA: 1, evpJ: 1, tssB: 1, tssC: 1, tssD: 0, tssE: 1, tssF: 1, tssG: 1, tssH: 0, tssI: 1, tssJ: 1, tssK: 0, tssL: 0, tssM: 1}
{tssA: 1, evpJ: 1, tssB: 1, tssC: 1, tssD: 0, tssE: 0, tssF: 1, tssG: 1, tssH: 1, tssI: 1, tssJ: 1, tssK: 1, tssL: 1, tssM: 1}
{tssA: 1, evpJ: 1, tssB: 1, tssC: 1, tssD: 0, tssE: 0, tssF: 1, tssG: 1, tssH: 1, tssI: 0, tssJ: 1, tssK: 1, tssL: 1, tssM: 1}
{tssA: 1, evpJ: 1, tssB: 0, tssC: 1, tssD: 1, tssE: 1, tssF: 1, tssG: 1, tssH: 1, tssI: 1, tssJ: 1, tssK: 1, tssL: 1, tssM: 1}
{tssA: 1, evpJ: 1, tssB: 0, tssC: 0, tssD: 1, tssE: 1, tssF: 0, tssG: 1, tssH: 1, tssI: 1, tssJ: 1, tssK: 0, tssL: 1, tssM: 0}
{tssA: 1, evpJ: 1, tssB: 0, tssC: 0, tssD: 1, tssE: 0, tssF: 1, tssG: 1, tssH: 0, tssI: 1, tssJ: 1, tssK: 1, tssL: 1, tssM: 0}
{tssA: 1, evpJ: 0, tssB: 1, tssC: 1, tssD: 1, tssE: 1, tssF: 1, tssG: 1, tssH: 0, tssI: 1, tssJ: 1, tssK: 1, tssL: 1, tssM: 1}
{tssA: 1, evpJ: 0, tssB: 1, tssC: 1, tssD: 1, tssE: 1, tssF: 0, tssG: 0, tssH: 0, tssI: 1, tssJ: 0, tssK: 1, tssL: 1, tssM: 0}
{tssA: 1, evpJ: 0, tssB: 1, tssC: 1, tssD: 1, tssE: 0, tssF: 0, tssG: 1, tssH: 1, tssI: 1, tssJ: 0, tssK: 1, tssL: 1, tssM: 1}
{tssA: 1, evpJ: 0, tssB: 1, tssC: 1, tssD: 1, tssE: 0, tssF: 0, tssG: 0, tssH: 1, tssI: 1, tssJ: 0, tssK: 1, tssL: 1, tssM: 1}
{tssA: 1, evpJ: 0, tssB: 0, tssC: 1, tssD: 1, tssE: 0, tssF: 1, tssG: 1, tssH: 1, tssI: 1, tssJ: 1, tssK: 1, tssL: 1, tssM: 1}
{tssA: 0, evpJ: 2, tssB: 0, tssC: 0, tssD: 0, tssE: 2, tssF: 1, tssG: 2, tssH: 0, tssI: 2, tssJ: 2, tssK: 1, tssL: 2, tssM: 0}
{tssA: 0, evpJ: 1, tssB: 1, tssC: 3, tssD: 2, tssE: 1, tssF: 1, tssG: 1, tssH: 1, tssI: 1, tssJ: 1, tssK: 1, tssL: 1, tssM: 1}
{tssA: 0, evpJ: 1, tssB: 1, tssC: 1, tssD: 1, tssE: 1, tssF: 1, tssG: 1, tssH: 1, tssI: 2, tssJ: 1, tssK: 1, tssL: 1, tssM: 1}
{tssA: 0, evpJ: 1, tssB: 1, tssC: 1, tssD: 1, tssE: 1, tssF: 1, tssG: 1, tssH: 1, tssI: 1, tssJ: 1, tssK: 1, tssL: 1, tssM: 1}
{tssA: 0, evpJ: 1, tssB: 1, tssC: 1, tssD: 1, tssE: 0, tssF: 1, tssG: 1, tssH: 1, tssI: 1, tssJ: 0, tssK: 1, tssL: 1, tssM: 1}
{tssA: 0, evpJ: 1, tssB: 1, tssC: 1, tssD: 0, tssE: 1, tssF: 1, tssG: 1, tssH: 0, tssI: 1, tssJ: 1, tssK: 1, tssL: 1, tssM: 1}
{tssA: 0, evpJ: 1, tssB: 1, tssC: 0, tssD: 1, tssE: 1, tssF: 0, tssG: 1, tssH: 0, tssI: 0, tssJ: 1, tssK: 1, tssL: 2, tssM: 1}
{tssA: 0, evpJ: 1, tssB: 0, tssC: 1, tssD: 1, tssE: 1, tssF: 1, tssG: 1, tssH: 1, tssI: 1, tssJ: 0, tssK: 0, tssL: 0, tssM: 0}
{tssA: 0, evpJ: 1, tssB: 0, tssC: 1, tssD: 1, tssE: 0, tssF: 1, tssG: 1, tssH: 1, tssI: 1, tssJ: 1, tssK: 1, tssL: 1, tssM: 1}
{tssA: 0, evpJ: 0, tssB: 1, tssC: 1, tssD: 1, tssE: 1, tssF: 1, tssG: 1, tssH: 1, tssI: 1, tssJ: 1, tssK: 1, tssL: 1, tssM: 0}
{tssA: 0, evpJ: 0, tssB: 1, tssC: 1, tssD: 1, tssE: 1, tssF: 1, tssG: 1, tssH: 1, tssI: 1, tssJ: 1, tssK: 0, tssL: 1, tssM: 1}
{tssA: 0, evpJ: 0, tssB: 1, tssC: 1, tssD: 1, tssE: 1, tssF: 1, tssG: 1, tssH: 0, tssI: 1, tssJ: 1, tssK: 1, tssL: 1, tssM: 0}
{tssA: 0, evpJ: 0, tssB: 1, tssC: 1, tssD: 1, tssE: 0, tssF: 0, tssG: 0, tssH: 1, tssI: 1, tssJ: 1, tssK: 1, tssL: 1, tssM: 1}
{tssA: 0, evpJ: 0, tssB: 0, tssC: 1, tssD: 1, tssE: 0, tssF: 1, tssG: 1, tssH: 0, tssI: 1, tssJ: 1, tssK: 1, tssL: 1, tssM: 1}
Operon organization
0
50
100
150
200
250
Number of systems

Figure S1

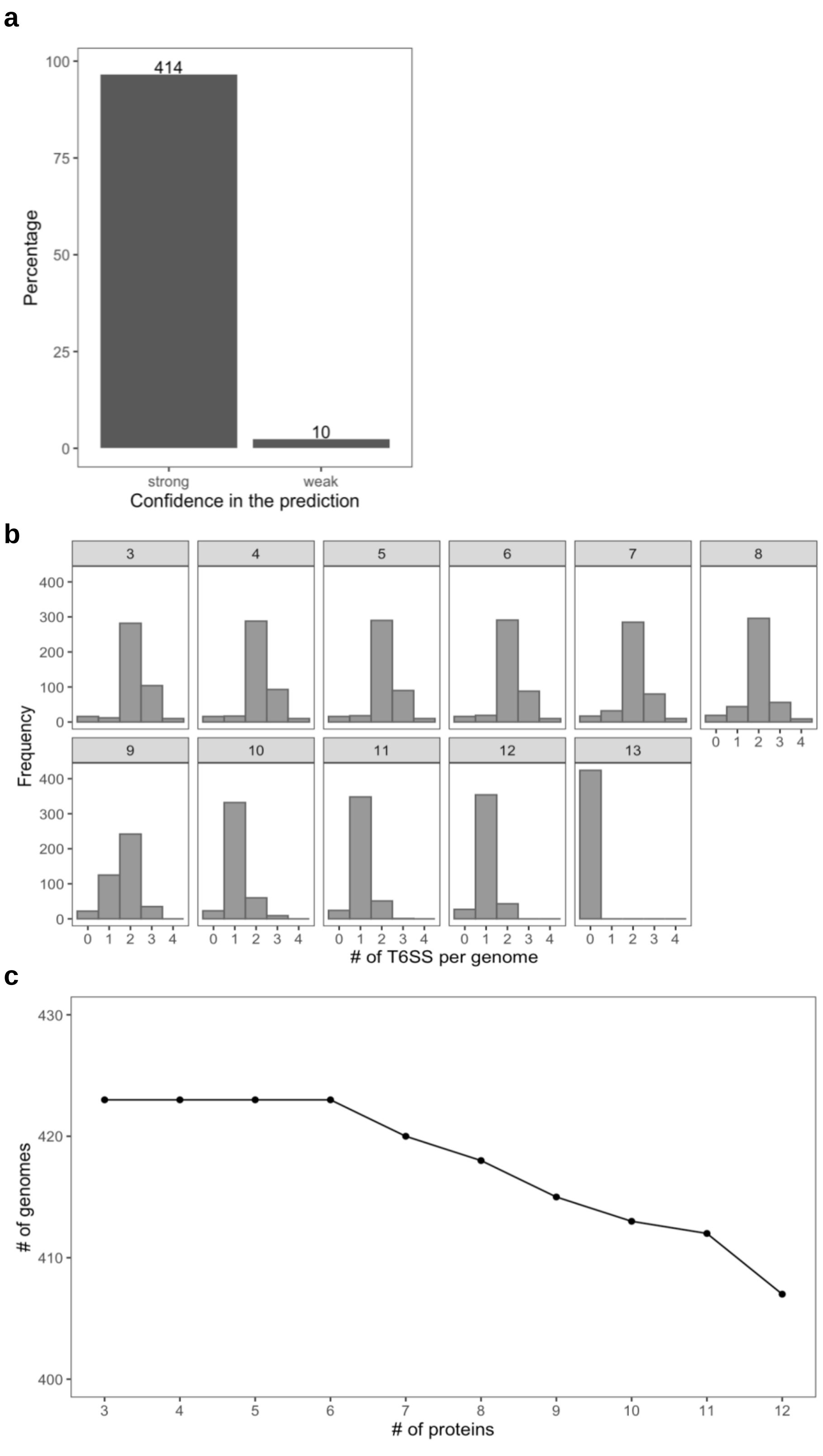

a
414
10
Percentage
100
75
50
25
0
strong
weak
Confidence in the prediction
b
3
4
5
6
7
8
9
10
11
12
13
Frequency
400
300
200
100
0
0 1 2 3 4
# of T6SS per genome
c
430
420
410
400
# of genomes
3 4 5 6 7 8 9 10 11 12
# of proteins

Figure S2

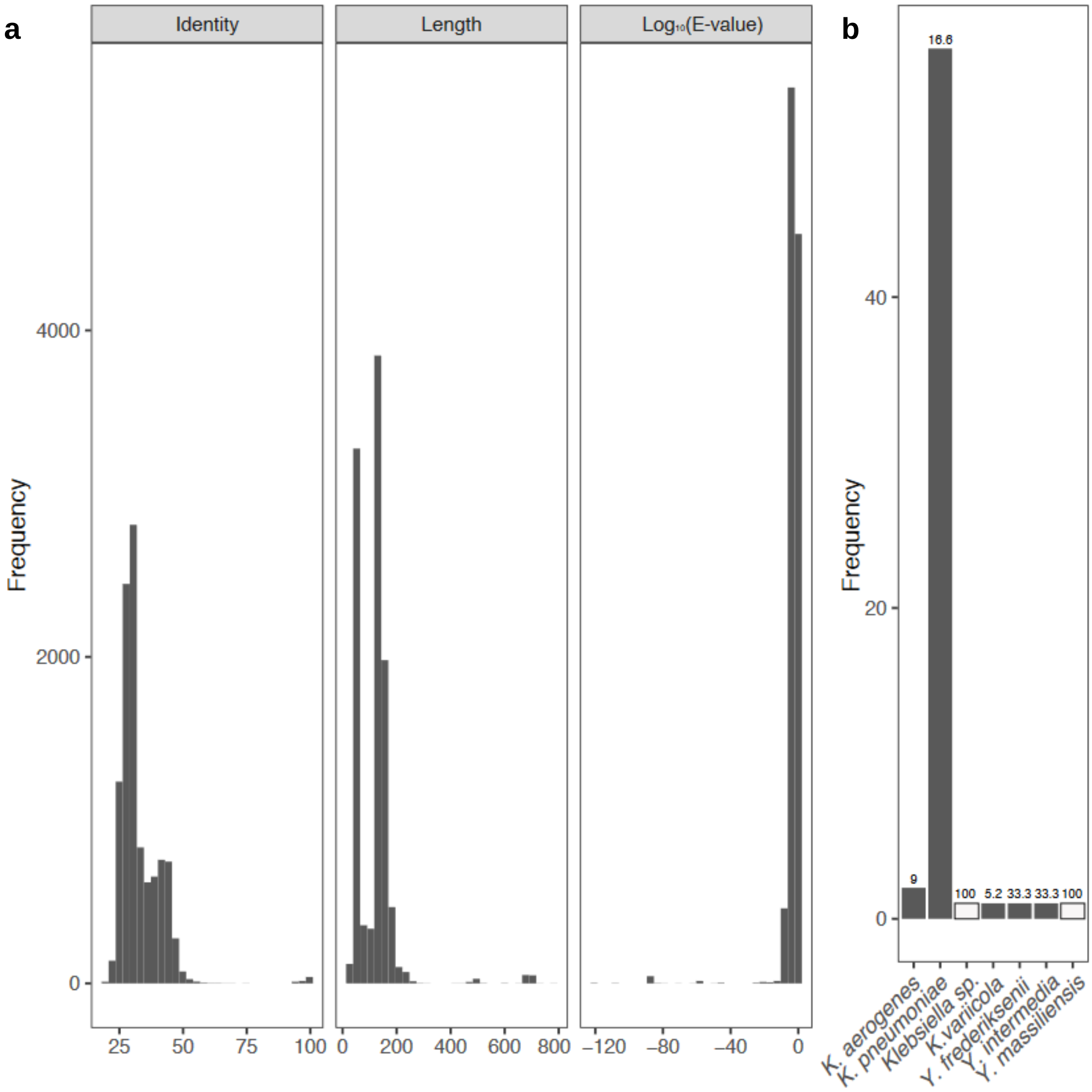
a
Identity
Length
Log₁₀(E-value)
Frequency
4000
2000
0
25
50
75
100
0
200
400
600
800
–120
–80
–40
0
b
16.6
9
100
5.2
33.3
33.3
100
40
20
0
K. aerogenes
K. pneumoniae
Klebsiella sp.
K.variicola
Y. frederiksenii
Y. intermedia
Y. massiliensis

Figure S3

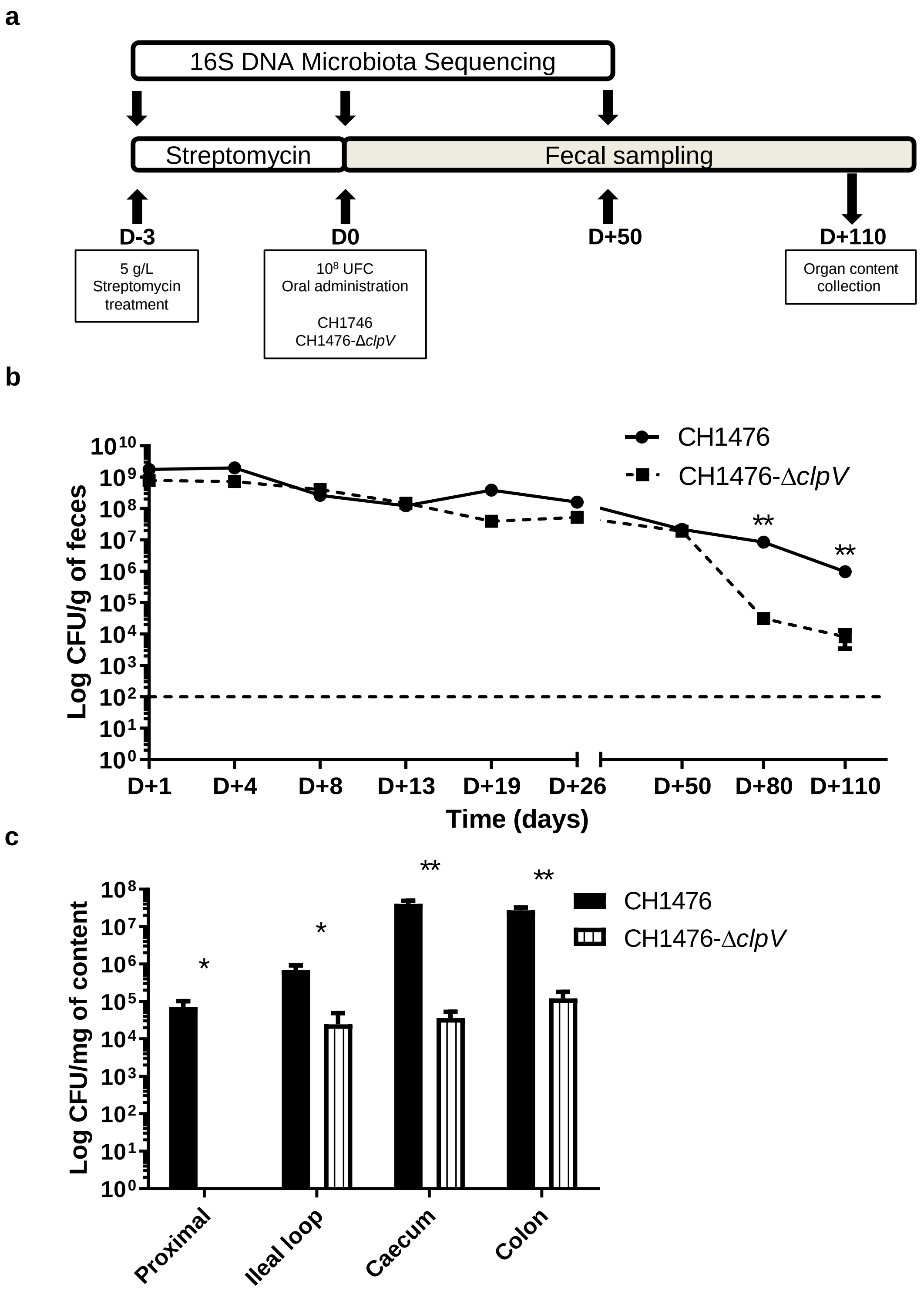
a
16S DNA Microbiota Sequencing
Streptomycin
Fecal sampling
D-3
D0
D+50
D+110
5 g/L Streptomycin treatment
10^8 UFC Oral administration
CH1746
CH1476-Δ*clpV*
Organ content collection
b
Log CFU/g of feces
10^{0} 10^{1} 10^{2} 10^{3} 10^{4} 10^{5} 10^{6} 10^{7} 10^{8} 10^{9} 10^{10}
D+1 D+4 D+8 D+13 D+19 D+26 D+50 D+80 D+110
Time (days)
CH1476
CH1476-Δ*clpV*
**
**
c
Log CFU/mg of content
10^{0} 10^{1} 10^{2} 10^{3} 10^{4} 10^{5} 10^{6} 10^{7} 10^{8}
Proximal
Ileal loop
Caecum
Colon
*
*
**
**
CH1476
CH1476-Δ*clpV*

Figure S4

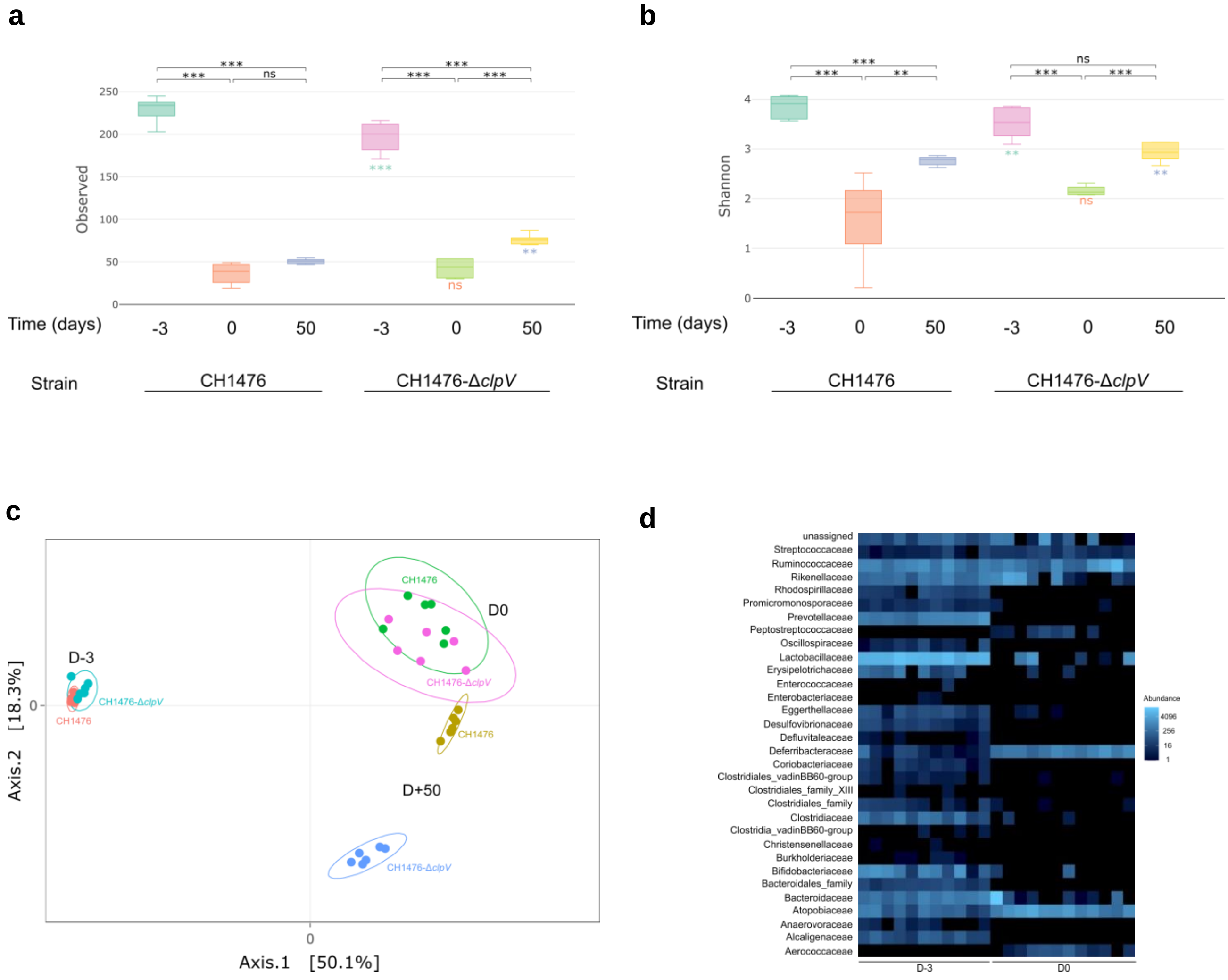

a
Observed
Time (days)
-3
0
50
Strain
CH1476
CH1476-ΔclpV
b
Shannon
c
D-3
D0
D+50
CH1476
CH1476-ΔclpV
Axis.1 [50.1%]
Axis.2 [18.3%]
d
Abundance
D-3
D0

Figure S5

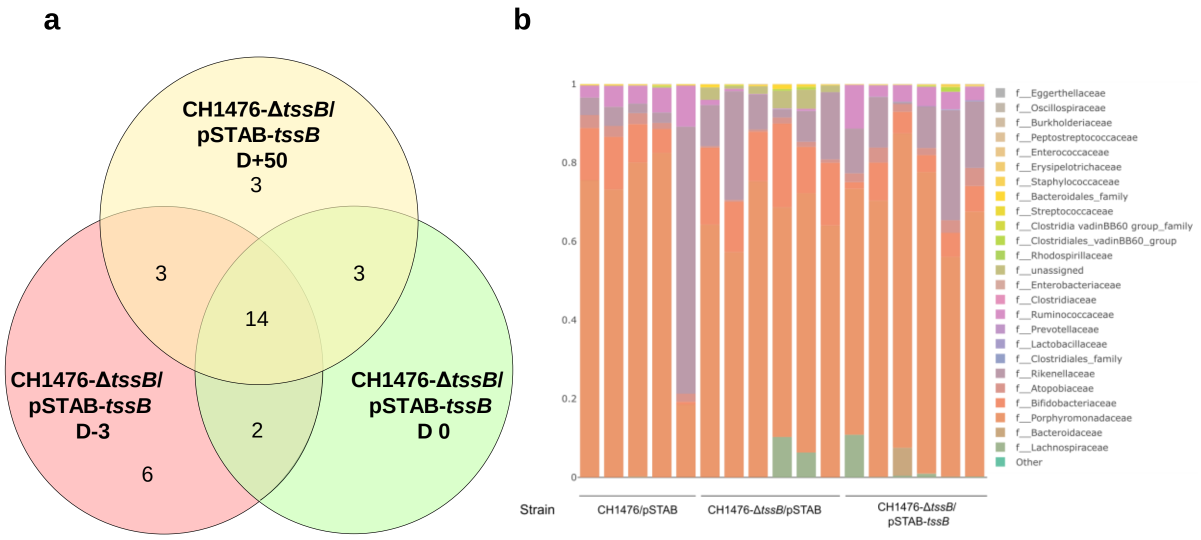
a
CH1476-Δ*tssB*/
pSTAB-*tssB*
D+50
3
3
3
14
2
6
CH1476-Δ*tssB*/
pSTAB-*tssB*
D-3
CH1476-Δ*tssB*/
pSTAB-*tssB*
D 0
b
1
0.8
0.6
0.4
0.2
0
Strain
CH1476/pSTAB
CH1476-Δ*tssB*/pSTAB
CH1476-Δ*tssB*/
pSTAB-*tssB*
f__Eggerthellaceae
f__Oscillospiraceae
f__Burkholderiaceae
f__Peptostreptococcaceae
f__Enterococcaceae
f__Erysipelotrichaceae
f__Staphylococcaceae
f__Bacteroidales_family
f__Streptococcaceae
f__Clostridia vadinBB60 group_family
f__Clostridiales_vadinBB60_group
f__Rhodospirillaceae
f__unassigned
f__Enterobacteriaceae
f__Clostridiaceae
f__Ruminococcaceae
f__Prevotellaceae
f__Lactobacillaceae
f__Clostridiales_family
f__Rikenellaceae
f__Atopobiaceae
f__Bifidobacteriaceae
f__Porphyromonadaceae
f__Bacteroidaceae
f__Lachnospiraceae
Other

Figure S6

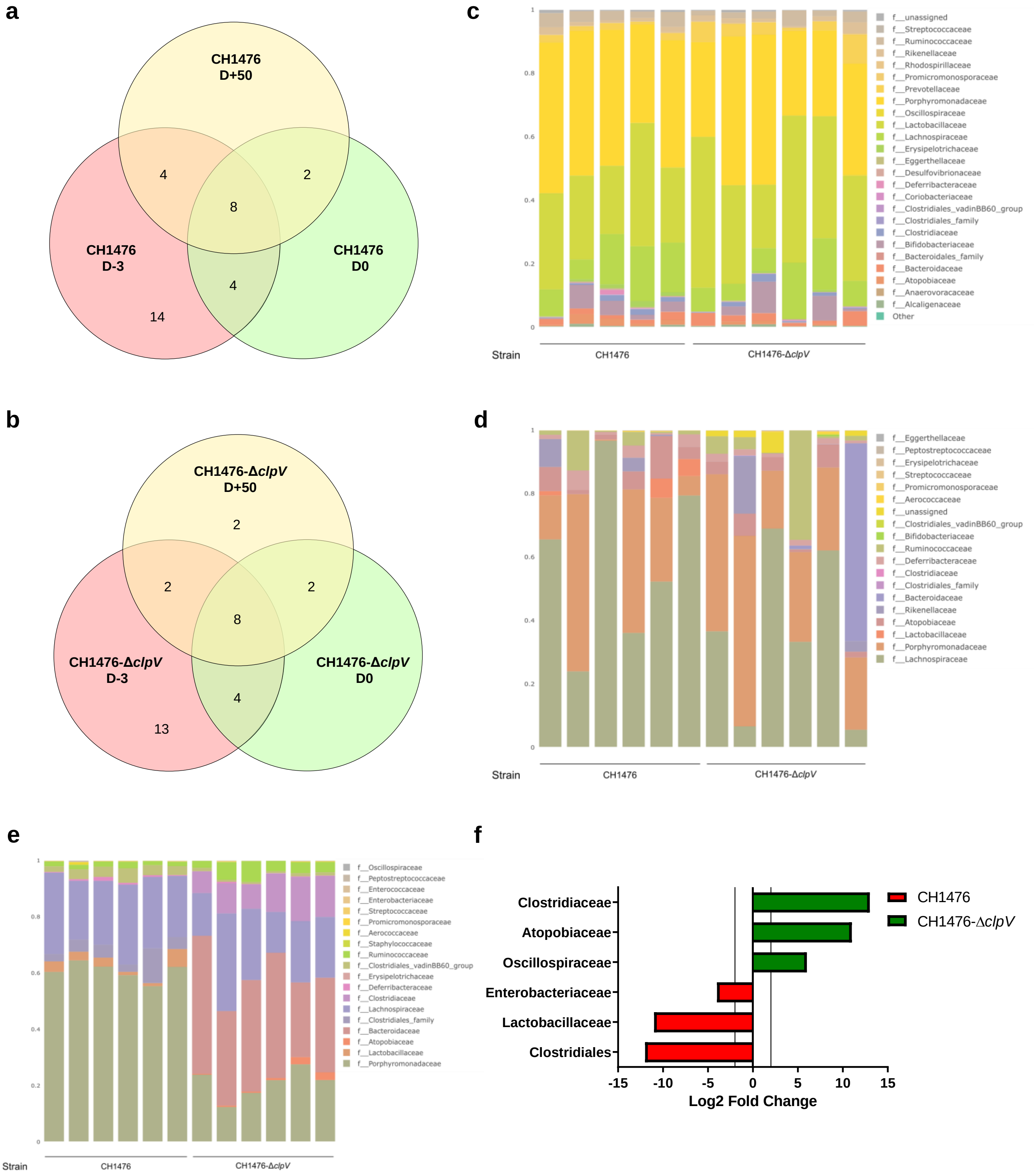
a
CH1476
D+50
CH1476
D-3
CH1476
D0
4
2
8
4
14
b
CH1476-ΔclpV
D+50
CH1476-ΔclpV
D-3
CH1476-ΔclpV
D0
2
2
2
8
4
13
c
Strain
CH1476
CH1476-ΔclpV
f__unassigned
f__Streptococcaceae
f__Ruminococcaceae
f__Rikenellaceae
f__Rhodospirillaceae
f__Promicromonosporaceae
f__Prevotellaceae
f__Porphyromonadaceae
f__Oscillospiraceae
f__Lactobacillaceae
f__Lachnospiraceae
f__Erysipelotrichaceae
f__Eggerthellaceae
f__Desulfovibrionaceae
f__Deferribacteraceae
f__Coriobacteriaceae
f__Clostridiales_vadinBB60_group
f__Clostridiales_family
f__Clostridiaceae
f__Bifidobacteriaceae
f__Bacteroidales_family
f__Bacteroidaceae
f__Atopobiaceae
f__Anaerovoracaceae
f__Alcaligenaceae
Other
d
Strain
CH1476
CH1476-ΔclpV
f__Eggerthellaceae
f__Peptostreptococcaceae
f__Erysipelotrichaceae
f__Streptococcaceae
f__Promicromonosporaceae
f__Aerococcaceae
f__unassigned
f__Clostridiales_vadinBB60_group
f__Bifidobacteriaceae
f__Ruminococcaceae
f__Deferribacteraceae
f__Clostridiaceae
f__Clostridiales_family
f__Bacteroidaceae
f__Rikenellaceae
f__Atopobiaceae
f__Lactobacillaceae
f__Porphyromonadaceae
f__Lachnospiraceae
e
Strain
CH1476
CH1476-ΔclpV
f__Oscillospiraceae
f__Peptostreptococcaceae
f__Enterococcaceae
f__Enterobacteriaceae
f__Streptococcaceae
f__Promicromonosporaceae
f__Aerococcaceae
f__Staphylococcaceae
f__Ruminococcaceae
f__Clostridiales_vadinBB60_group
f__Erysipelotrichaceae
f__Deferribacteraceae
f__Clostridiaceae
f__Lachnospiraceae
f__Clostridiales_family
f__Bacteroidaceae
f__Atopobiaceae
f__Lactobacillaceae
f__Porphyromonadaceae
f
Clostridiaceae
Atopobiaceae
Oscillospiraceae
Enterobacteriaceae
Lactobacillaceae
Clostridiales
-15 -10 -5 0 5 10 15
Log2 Fold Change
CH1476
CH1476-ΔclpV

Figure S7
